# In silico predicted compound targeting the IQGAP1-GRD domain selectively inhibits growth of human acute myeloid leukemia

**DOI:** 10.1038/s41598-024-63392-2

**Published:** 2024-06-04

**Authors:** Deepak M. Sahasrabudhe, Jane L. Liesveld, Mohammad Minhajuddin, Niloy A. Singh, Subhangi Nath, Vishuwes Muthu Kumar, Marlene Balys, Andrew G. Evans, Mitra Azadniv, Jeanne N. Hansen, Michael W. Becker, Ashoke Sharon, V. Kaye Thomas, Richard G. Moore, Manoj K. Khera, Craig T. Jordan, Rakesh K. Singh

**Affiliations:** 1 Wilmot Cancer Institute, University of Rochester Medical Center, 601 Elmwood Avenue, Box 704, Rochester, NY 14618 USA; 2 Department of Medicine, Hematology/Oncology, University of Rochester Medical Center, Rochester, NY USA; 3 Division of Hematology, University of Colorado Anschutz Medical Campus, Aurora, Colorado US; 4 Department of Chemistry, Birla Institute of Technology, Ranchi, Jharkhand India; 5 Genomics Research Center, University of Rochester Medical Center, Rochester, NY USA; 6 Department of Pathology and Laboratory Medicine, University of Rochester Medical Center, Rochester, NY USA; 7 Department of Psychological and Brain Sciences, Colgate University, Hamilton, NY USA; 8 Indiana University School of Medicine, Indianapolis, IN USA; 9 Department of Pharmacology and Physiology, University of Rochester Medical Center, Rochester, NY 14642 USA; 10 Division of Obstetrics and Gynecology, University of Rochester Medical Center, Rochester, NY USA; 11 Presude Lifesciences Pvt Ltd., Uttam Nagar, New Delhi, 110059 India; 12 Wilmot Cancer Institute, University of Rochester Medical Center, 601 Elmwood Avenue, Rochester, NY 14642 USA

**Keywords:** Cancer, Cell biology, Drug discovery, Immunology, Oncology

## Abstract

Acute myeloid leukemia (AML) is fatal in the majority of adults. Identification of new therapeutic targets and their pharmacologic modulators are needed to improve outcomes. Previous studies had shown that immunization of rabbits with normal peripheral WBCs that had been incubated with fluorodinitrobenzene elicited high titer antibodies that bound to a spectrum of human leukemias. We report that proteomic analyses of immunoaffinity-purified lysates of primary AML cells showed enrichment of scaffolding protein IQGAP1. Immunohistochemistry and gene-expression analyses confirmed IQGAP1 mRNA overexpression in various cytogenetic subtypes of primary human AML compared to normal hematopoietic cells. shRNA knockdown of IQGAP1 blocked proliferation and clonogenicity of human leukemia cell-lines. To develop small molecules targeting IQGAP1 we performed *in-silico* screening of 212,966 compounds, selected 4 hits targeting the IQGAP1-GRD domain, and conducted SAR of the ‘fittest hit’ to identify UR778Br, a prototypical agent targeting IQGAP1. UR778Br inhibited proliferation, induced apoptosis, resulted in G2/M arrest, and inhibited colony formation by leukemia cell-lines and primary-AML while sparing normal marrow cells. UR778Br exhibited favorable ADME/T profiles and drug-likeness to treat AML. In summary, AML shows response to IQGAP1 inhibition, and UR778Br, identified through *in-silico* studies, selectively targeted AML cells while sparing normal marrow.

## Introduction

Acute myeloid leukemia (AML) is often a clinically aggressive disease that is fatal in the majority of patients^1^. AML has emerged as the leading cause of leukemia-related deaths in the US^2^. While outcomes of pediatric AML have improved significantly^3^, AML remains a major clinical concern particularly among older patients^4^ and those with relapsed disease^5–8^Despite introduction of new therapies, the 5-year survival remains below 30%. It appears that the efficacy of currently available treatments and their combinations may have reached a plateau^9–11^. Therefore, identification of new driver-like therapeutic targets and development of pharmacologic modulators that can specifically block their function in AML while sparing normal progenitors, i.e. possessing a favorable therapeutic index, is urgently needed to improve prognosis^11,12^.

In order to identify a shared antigenic moiety that could serve as a druggable target for novel therapeutics in AML, we replicated previously published experiments which had shown that immunization of rabbits with human peripheral blood white blood cells (WBCs) pre-incubated with fluorodinitrobenzene (FDNB) elicited high titer antibodies that agglutinated peripheral blood white cells from a broad spectrum of human leukemia, including AML, chronic myeloid leukemia (CML), acute lymphocytic leukemia (ALL) and chronic lymphocytic leukemia (CLL)^13,14^. A summary of previously published data is presented in the supplementary section as an Addendum. We report that this approach identified IQGAP1, a scaffolding protein, as a shared antigenic moiety in human AML.

IQGAP1 is a multi-domain, cytoskeletal protein, shown to be upregulated in many solid tumors^15–17^. IQGAP1consists of five functional domains: a calponin-homology domain (CHD, 44–159 aa), a poly-proline protein–protein domain (WW, 681–710 aa), a domain containing four IQ-motif (IQ, 745–864 aa), a Ras GAP-related domain (GRD, 1004–1237 aa), and a Ras GAP C-terminal domain (RGCT 1276–1657 aa); through which it binds to other proteins. Through these binding domains IQGAP1 interacts with a large number of protein binding partners including MAPK, RAC1/CDC42, WNT, PI3K, Hippo and TGF-β. Acting as a scaffolding protein, IQGAP1 participates in various biological activities, including signal transduction, cytoskeletal dynamics, cytokinesis, cell polarizations, cadherin mediated cell–cell adhesion, cell polarization and actin reorganization to promote cell migration, invasion, and cell proliferation, many of which are involved in tumorigenesis^15–17^.

However, the role of IQGAP1 has not been examined in AML. Similarly, small molecules targeting IQGAP1 with therapeutic potential for treatment of AML and other malignancies were not available when we undertook the work. We report that immunization of rabbits with peripheral blood WBCs preincubated with FDNB followed by proteomic analyses of immunoaffinity purified lysates of primary AML detected enrichment of IQGAP1. Immunohistochemistry and gene-expression analyses confirmed IQGAP1 mRNA overexpression in various cytogenetic subtypes of primary human AML compared to normal hematopoietic cells. shRNA knockdown of IQGAP1 blocked proliferation and colony formation in human leukemia cell-lines. To identify a small molecule targeting IQGAP1, in silico screening of a large library of structurally diverse compounds followed by structure–activity relationship (SAR) guided optimization of the preferred hit was undertaken to select UR778Br, a potent and first-in-field small molecule modulator of the IQGAP1-GRD domain. To gain insight in the mechanism of action of UR778Br against cytoskeletal assembly of IQGAP1/F-actin^18^, we employed confocal microscopy (data not shown). To evaluate therapeutic potential of UR778Br, its effect against viability, colony formation potential and cell cycle progression of AML versus normal marrow cells were examined. Taken together, the data show that IQGAP1 is a potential therapeutic target in AML. A small molecule, UR778Br, targeting IQGAP1, has been identified which has preferential activities against AML while sparing normal marrow.

## Materials and methods

### AML cell lines, primary samples and antibodies used

K562, KG1α, MOLM13, MV411, THP1 and U937 cells procured from ATCC were cultured in RPMI-1640 medium supplemented with 10% fetal bovine serum (FBS) (Hyclone) and 1% penicillin/streptomycin. Antibodies used were anti-IQGAP1 murine monoclonal (D3): (Santa Cruz Biotechnology Inc. catalog number: sc-374307), anti-IQGAP1 rabbit monoclonal (D6E3J) (Cell Signaling Technology, catalog number: 290165), anti-GAPDH rabbit monoclonal (Cell Signaling Technology, catalog number: 14C10), anti-α tubulin rabbit monoclonal (Cell Signaling Technology, catalog number: 11H10), anti-cleaved PARP (Asp214) rabbit monoclonal antibody (Cell Signaling Technology, catalog number: 5625), HRP-conjugated anti-rabbit antibody (Cell Signaling Technology, catalog number: 7074), and HRP-conjugated anti-mouse antibody (Cell Signaling Technology, catalog number: 7076). UR778Br, off-white to pale-yellow solid, was contract manufactured by Toronto Research Chemicals (TRC, lot number:35-AZC-183-1) and by Presude Lifesciences Private Limited (India). Presude also resolved the individual isomers (R- and S-). Purity: > 95%. Elemental analysis: %C:57.79, %H:4.88, %N:3.33. NMR and Mass spectrum conformed to the structure; Specific Rotation (SOR):0.0^o^ (c = 0.5. DMSO).

### Peripheral blood and bone marrow

The protocol for collecting peripheral blood samples from healthy volunteers was approved by the University of Rochester’s Research Subject Review Board. All healthy donors provided informed consent. De-identified samples of AML and normal/healthy donors that had been collected under a protocol approved by the Research Subject Review Board were used in immunoaffinity purification experiments, Western blot and IHC. The collection of peripheral blood samples from healthy volunteers and de-identified AML samples were done in compliance with institutional, local and national guidelines.

### Immunization of rabbits

The research protocol for immunizing rabbits and obtaining blood samples was approved by the University of Rochester’s Institutional Animal Care and Use Committee. The rabbits were housed in an AAALAC accredited vivarium overseen by the centralized Animal Resource management. The rabbit room was maintained on a 12:12 light cycle (lights on 0600), at 66.5 ± 2°F, 30–70% humidity and experienced 10–15 air changes/hour. These environmental parameters were continuously monitored electronically and remained within NIH set points. Rabbits were housed in stainless steel and plastic molded cages providing sufficient space in compliance with Animal Welfare regulations. Rabbits received *ad lib* food and water during the entire study. Rabbits were also given enrichment manipulation as described in the University of Rochester’s Enrichment Plan for Laboratory Animals. Animal care technicians provided daily husbandry services and reported animal illness, injury or other abnormal behavior to the Division of Laboratory Animal Medicine (DLAM) veterinary staff. The immunization, blood draws and euthanasia of rabbits were performed following IACUC approved protocol by NYS licensed veterinary technicians and veterinarians (DLAM). Immunization of rabbits, collection of blood samples and euthanasia were performed in compliance with institutional, local and national guidelines. The rabbits were sedated for blood collection from the central ear artery. Sedation was accomplished using a combination of droperidol (2.5 mg/kg IM) and Fentanyl (0.05 mg/kg IM). The rabbits were placed in a restraint box. No more than 10–15% of the rabbit's blood volume (6–9 ml/kg body weight) was drawn at one time.

Rabbits were euthanized at the last bleed by cardiac exsanguination under a surgical plane of anesthesia (achieved by ketamine 44 mg/kg IM and xylazine 5 mg/kg IM). Death confirmed by observation of cessation of voluntary movements and auscultation of the chest for absence of a heartbeat and respiration. In the event of a rabbit becoming ill prior to experimental endpoint, and in the judgement of the investigator and veterinarian, euthanasia were warranted, pentobarbital 100 mg/kg IV was to be administered in accordance with the AVMA Guidelines for Euthanasia (June 2007).

This study is reported in accordance with ARRIVE guidelines.

### Isolation of peripheral blood WBCs and incubation with fluorodinitrobenzene

Blood from healthy volunteers was drawn by venipuncture into acid citrate dextran containing tubes. Collected blood was diluted with equal volume of phosphate buffered saline, without calcium or magnesium, pH 7.2. Red blood cells were sedimented by adding 6 ml of 6% dextran (Molecular weight 500,000, cat #9605D, Lot# A84991, Research Organics, Cleveland, OH) in PBS to 40 ml of the diluted blood and incubating at room temperature for 20 min. The supernatant containing WBCs was aspirated and centrifuged. Contaminating RBCs were removed by hypotonic shock lysis. Cell count was determined by counting in a hemocytometer and viability was determined by Trypan blue dye exclusion. Viability was determined to be more than 98%. 2,4-dinitrofluorobenzene (FDNB) (Sigma-Aldrich, catalog number: D1529) at 1.482 g/mL was used. As FDNB is photosensitive and highly reactive the dilutions and incubation of cells was carried out in glass tubes and exposure to fluorescent light was avoided. FDNB was serially diluted to 31 picograms/mL. Based on Avogadro’s number this concentration corresponds to 1 × 10^11^ molecules per mL. WBCs at 1 × 10^7^ cells/mL were mixed with equal volume of FDNB at 31 picograms corresponding to 1 × 10^11^ molecules per ml, yielding an average of 1 × 10^4^ molecules per cell. The cells were incubated for 12–15 min at room-temperature, washed, re-suspended in ice cold PBS, counted and 1 × 10^7^ cells were injected subcutaneously, once per week for 16 weeks in three rabbits. A rabbit was injected with an equal number of unmodified WBCs subcutaneously once per week for 16 weeks as the control. At week 16 blood samples were collected by venipuncture, serum separated, complement was inactivated, and the sera were absorbed against pooled unmodified WBCs from normal healthy volunteers at 37 °C for 1 h. The absorbed sera were centrifuged and aliquots were stored at − 80 °C. After confirming the presence of an immune response, the rabbits were euthanized. Sera were separated, complement inactivated by heating the sera at 56 °C for 60 min and aliquots stored at − 80 °C.

### Immunoaffinity purification

Protein A/G PLUS Agarose Beads (Santa Cruz Biotechnologies, catalog number: sc-2003) were used per manufacturer’s instructions. We adopted a strategy of depletion followed by enrichment. Cell pellets were lysed in radioimmunoprecipitation (RIPA) buffer (50 mM Tris–HCl, pH 7.5, 150 mM NaCl, 1% deoxycholic acid, 1% Triton X-100, 0.25 mM EDTA, and 5 mM sodium fluoride) to which Halt Protease and Phosphatase inhibitor cocktail (Thermo Scientific, catalog number: 78440) was added. One hundred microliters of the lysis buffer were added per million cells. The pellets were disaggregated by passing through a 21G needle × 5 and were incubated on ice for 15 min and centrifuged at 13000 rpm for 10 min. Supernatant was collected and the protein concentration was measured using Piercenet BCA following manufacturer’s instruction. Similarly, whole cell lysates of de-identified clinical AML samples were incubated with protein A/G agarose beads to which IgG from the rabbit immunized with unmodified WBCs was attached. An aliquot of the unbound fraction was set aside for protein estimation, Western blot and for IP. The remainder of the unbound fraction was incubated overnight at 4ºC with protein A/G agarose beads to which IgG from rabbit immunized with *WBCs modified by FDNB* was attached. An aliquot of the unbound fraction was set aside for protein estimation, Western blot and for IP. Antigens bound to agarose beads were eluted by boiling the beads.

### Proteomic analysis

For LC–MS/MS analysis the three samples: index-, affinity purified- or depleted protein samples were cleaned up for lipid and detergent removal by a 10 min electrophoresis step on SDS-PAGE. The gel was stained with Coomassie blue and the gel portions were isolated, processed for trypsin digestion and LC–MS/MS analysis. In brief, gel pieces were diced into 1-mm squares, rinsed with water and 50 mM ammonium bicarbonate buffer, and dehydrated. Reduction of disulfide bonds was conducted with 10 mM dithiothreitol, followed by alkylation with 50 mM iodoacetamide. Proteins were digested by rehydrating the gel slices in 20 μg/ml trypsin (Promega) in ammonium bicarbonate buffer plus 10% acetonitrile for 1 h at 24 °C, followed by overnight incubation at 37 °C and a second addition of trypsin the next day for 3 h. The digested material was extracted from the gel, combined, and dried, using a vacuum concentrator. 10–20% of the digest was loaded on a Magic C18 AQ (Michrom) Nano spray column/tip on a Thermo LTQ mass spectrometer and washed with 5% methanol, 0.1% formic acid for 10 min before peptide elution began, using a 5–60% methanol gradient. The LTQ ion trap mass spectrometer, equipped with a nano electrospray ionization source, ran a full MS survey scan every 3 s in the data-dependent mode to collect seven MS/MS fragmentation spectra per cycle, using dynamic exclusion to access lower intensity peptides. The raw data files containing MS and fragmentation spectrum data were used in a Mascot search, against the complete human and rabbit proteomes. Mascot (Matrix Sciences) search parameters included precursor and fragment ion mass tolerance of 1.5 and 0.8 Daltons, respectively, one 13C incorporation, one missed trypsin cleavage site, fixed carbamidomethyl-cysteine modification, and variable methionine oxidation. Mascot search results files were uploaded into Scaffold (Proteome Software) and additionally searched with X!Tandem (X!Tandem-The GMP) in the Scaffold environment. Threshold values for protein identification were 95% minimum protein identification probability, with 90% peptide probability. Relative abundance of a protein in different samples was semi-quantitatively determined, using spectral counting analysis.

### IQGAP1 mRNA expression in primary human AML and normal HSC controls and survival analysis

**Figure 1:**
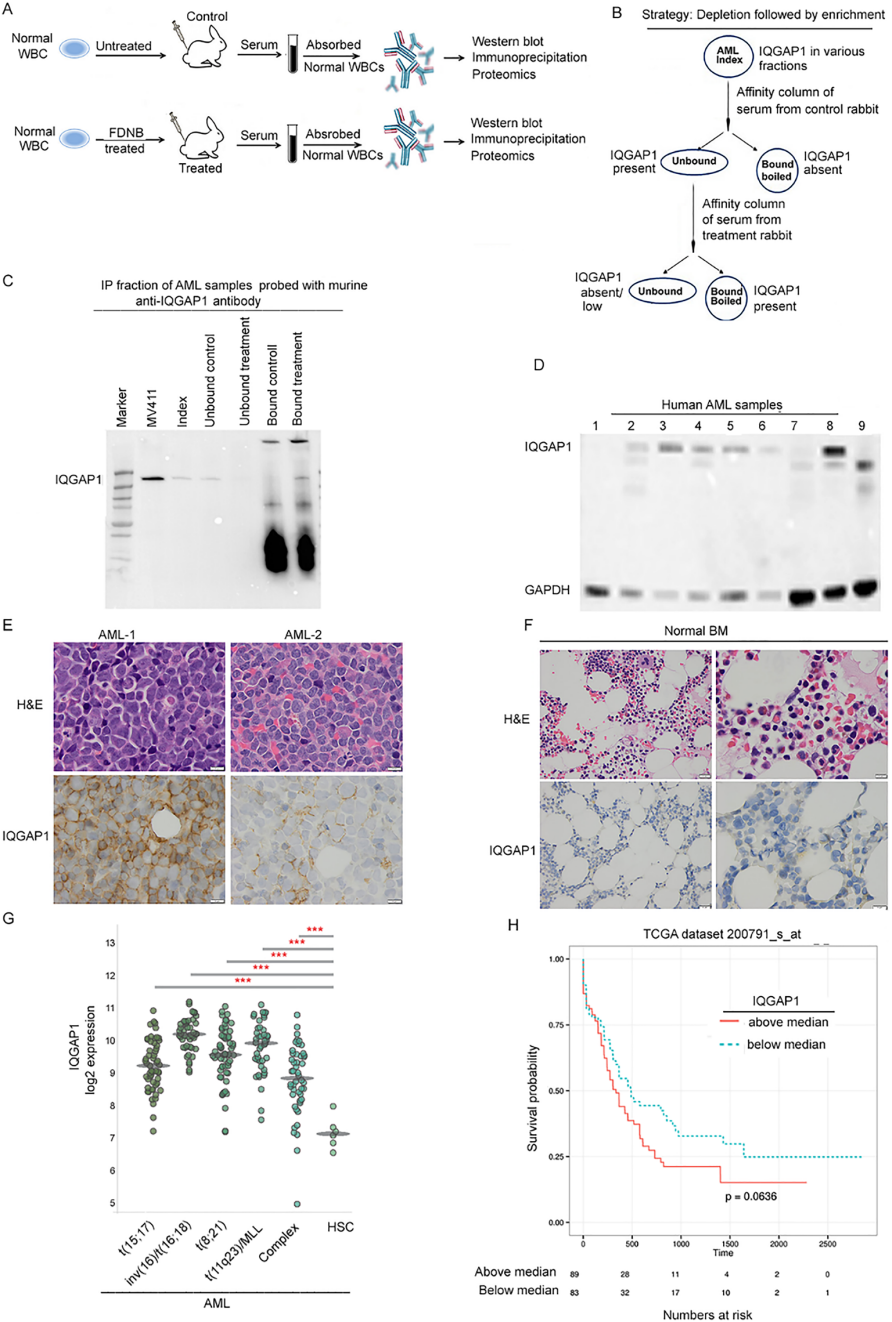
(**A**). General overview of experimental design**:** Normal WBCs exposed to FDNB or vehicle were injected subcutaneously into rabbits; serum collected, complement inactivated and absorbed against normal WBCs and analyzed by western blots and immunoprecipitation and proteomics to isolate the antigenic moiety. (**B**): Strategy of depletion followed by enrichment**.** Whole cell lysates of de-identified clinical AML samples were incubated with protein A/G agarose beads to which IgG from the rabbit immunized with untreated WBCs was attached. An aliquot of the unbound fraction was set aside for protein estimation, western blot and for IP. The remainder of the unbound fraction was incubated overnight at 4 °C with protein A/G agarose beads to which IgG from rabbit immunized with FDNB-treated WBCs was attached. An aliquot of the unbound fraction was set aside for protein estimation, Western blot and for IP. Antigens bound to agarose beads were eluted by boiling the beads. LC–MS/MS analyses of the three samples were performed: 1. unbound to A/G beads with IgG from rabbit immunized with untreated WBCs, 2. eluted from A/G beads with IgG from rabbit immunized with untreated WBCs, and 3. eluted from A/G beads with IgG from rabbits immunized with FDNB treated WBCs. Proteins that were shared between fraction 1 and 3 and not present in fraction 2 were deemed to be of interest. (**C**): After identifying IQGAP1 as the component of interest in the antigenic moiety, western blot of the five fractions: index whole cell lysate of the AML and the fractions that did not bind to and ones that were eluted off the affinity columns sequentially were probed with a murine anti-IQGAP1 antibody. Whole cell lysate of MV411 was used as a positive control. (**D**): Whole cell lysates of nine de-identified primary AML samples probed with a murine anti-IQGAP1 antibody and rabbit anti-beta actin antibody. (**E**): H&E and IHC for IQGAP1 in two primary AML samples and (**F**) two normal bone marrow samples show that IQGAP1 is overexpressed in the two primary AML samples compared to two normal bone marrow samples. (**G**)**:** Determination of IQGAP1 mRNA expression in primary human AML and normal HSC controls**:** Analysis of the gene expression in the Bloodspot database (http://servers.binf.ku.dk/bloodspot/) revealed increased expression of IQGAP1 in human AML cytogenetic subtypes than in normal hematopoietic stem cell (HSC) controls, *p* < 0.001 students T test. (**H**) IQGAP1 overexpression above the median cut-off trends toward poor prognosis (*p* = 0.063).

Expression of IQGAP1 in AML sub-types versus HSCs (Fig. 1G) and survival analysis (Fig. 1H) of AML patients based on above/below median expression levels of IQGAP1 protein was performed using Bloodspot portal. To determine the expression of IQGAP1 in a larger sample the raw data from the publication by Gentles^6^ were downloaded from the Gene Expression Omnibus (GEO) at the National Center for Biotechnology Information (NCBI) and is available under accession number GSE24006. Data analysis was carried out using Partek Genomic Suite (Partek Inc. Saint Louis, MO). Chips were normalized using Robust Multi-array Average (RMA) and a one-way ANOVA was conducted to interrogate significance on the IQGAP probe set "213446_s_at.".

### Proliferation

To measure proliferation, K562, MV411 and THP1, naive cells and cells transfected with two different shRNA targeting IQGAP1 as well as transfected with scrambled shRNA were dispersed in 48-well plates at 50,000 or 100,000 cells per ml. Cell counts were done by hemocytometer on days 1, 2, and 3. Each experiment was repeated three times. To measure the effect of UR778Br on the proliferation MOLM13, MV411, THP1 and U937 cells were seeded at 100,000 cells/ml density per well in triplicate in 96-well plates. UR778Br was added to achieve indicated concentrations. DMSO was added as a control. Cell counts were performed on hemocytometers in triplicate on days 1, 2, and 3.

### shRNA knockdown of IQGAP1

IQGAP1 expression in K652, MV411 and THP1 cells was transiently knocked down via shRNAs oligos. The indicated cell lines were transfected with shRNA targeting IQGAP1 or scrambled shRNA using Lipofectamine 3000 (Invitrogen). shRNA sequences:

### sh-IQGAP1-Clone-B

Forward 5′- ccggGCATCCACTTACCAGGATATActcgagTATA TCCTG GTAA GTGGATGCtttttg -3′; and reverse 3’- aattcaaaaaGCATCCACTTACCAGGATATActcgagTATATC CTGGTAAGTGGATGC- 5’ and sh-

### IQGAP1-Clone C

Forward 5′- ccggGCAGCTCCTGAGTGATAAACAct cgagTGTTTATCACTCAGGAGCTGCtttttg -3′ and reverse 3’- aattcaaaaaGCAGCTCCTGAGTGATAAAC ActcgagTGTTTATCACTCAGGAGCTGC—5’.

### MMO17scramble-pLKO

Forward:5’-ccggGCCTAAGGTTAAGTCGCCCTCGctcgagCGAGGGCGACTTAACCTTAGGtttttg- 3’; reverse: 5’ aattcaaaaaCCTAAGGTTAAGTCGCCCTCGctcgagCGAGGG CGACTTAACCTTAGG -3’.

Cells were incubated for 72 h after transfections, harvested by centrifugation and lysed and immunoblotted using IQGAP1 primary antibody (Santa Cruz Biotechnology, catalog number: sc-374307). GAPDH antibody (Santa Cruz Biotechnology, catalog number: sc-47724) was used as the loading control.

### Western blot analysis of primary AML cells

De-identified clinical AML samples collected under an IRB-approved protocol were used for Western blot and immunoaffinity purification. An aliquot of the lysate was mixed with equal volume of 2X Laemmle buffer plus 2 beta-mercaptoethanol, boiled for 10 min and then stored at − 20 °C. The samples were electrophoresed in 10% triglycine gels (Bio-Rad), resolved proteins were transferred to PVDF membrane (Immobilon, Millipore), blocked with 5% dry milk in tris-buffered saline with 0.5% Tween for 1 h at room temperature, and probed with immune sera from the rabbits at 1:5000 dilution at 4 °C overnight. Horseradish peroxidase conjugated anti-rabbit antibody (Cell Signaling) as the secondary antibody and Amersham ECL Prime reagent (GE Healthcare Biosciences) or Super Signal West Femto Maximum Sensitivity Substrate, (Thermo Scientific, catalog number: 34095) were used. The blots were read using BioRad ChemiDoc MP Imaging System and analyzed with the provided software.

### Virtual screening

The complex structure of Ras/GAP-334 with PDB code of 1WQ1^19^ was used to obtain the relative position of GRD and Cdc42. Specifically, the structures of GRD (PDB code 3fay) and Cdc42 (PDB code 1AN0) were superimposed onto GAP-334 and Ras, respectively. The binding model of GRD with Cdc42 was previously described^21^. The conserved motif 1192YYR1194 was located inside the protein binding interface. T1046 is positioned near the GDP from Cdc42, which is consistent with the model described in the literature^21^. The region around 1192YYR1194 was chosen as a binding site for virtual screening. The 3D protein structure file (.pdb) was retrieved from the Protein Data Bank (PDB) with the accession code 3fay. Water molecules as well as other irrelevant ligands were removed from the pdb file, while hydrogen atoms were added to the protein. Energy minimization was done to remove the clashes. The SD file of specs compounds were downloaded from the website www.specs.net. A molecular weight filter was used to keep the molecules within 100–800. The molecules were then converted from 2 to 3D structure and energy minimized using OpenBabel 2.3.0. Virtual screening was performed by using AutoDock Vina. Receptor and ligands were prepared using MGLTools 1.5.6. Polar hydrogen atoms were added and gasteiger charges were assigned for all the ligands. AutoDock generates different ligand conformers using a Lamarckian genetic algorithm (LGA). The center of the grid box was set to the center of R1194. The box size was set to 80 × 80 × 80 with grid spacing 0.375 Å in each dimension, which is large enough for the free rotation of the ligand. All the other parameters were kept as default. The compounds from specs.net (www.specs.net) were used as ligands in the virtual screening. Only molecules with molecular weight larger than 100- and less than 800 gm/mol were used. A total of 212,966 molecules entered the virtual screening experiment.

### Computational methodology of docking AK778 analogs

The crystal structure of the GAP-related domain of IQGAP1 (PDB ID: 3FAY) was imported. The crystal structure of the GAP-related domain (GRD) of 1QGAP1 (PDB ID: 3FAY) was downloaded from RCSB PDB database and imported to Schrödinger Suite 2021. The protein structure^21^ specifies the domain which is homologous to the Ras GTPase-activating protein domain. Therefore, the PDB ID: 3FAY reports the crystal structure of the isolated IQGAP1 GRD whereas TRS (Tris buffer) indicates the coordinates deposited in the database to specify the GRD domain. The imported protein structure was prepared and refined in the protein preparation wizard of Maestro, Schrödinger^22^. This step is important in order to ensure assignment of correct bond orders, addition of hydrogens, addition of missing residues, and addition of missing loops using Prime. All water molecules beyond 5.0 Å were deleted. Het states were generated using the Epik tool at pH 7.0 ± 2.0. The prepared protein structure was minimized with RMSD constraint of 0.3 Å using OPLS_2005 force field. SiteMap^23^ is used to investigate binding sites of the protein structure. The probable binding sites are ranked on the basis of SiteScores and Dscores. These scores take into various factors such as size, volume, hydrophobicity and hydrophilicity. The Dscores give the drugability of the binding sites. For sitemap, fine grid was marked. The rest of the parameters were set according to the default settings. The 2D structures of the ligands were drawn in ChemDraw 2015 and imported their 3D structure to the Maestro workspace. The structures were minimized using MacroModel^24^ module using OPLS_2005 with 10,000 iterations. The job was run with default settings. The ligands were prepared using Ligprep^25^ tool in order to generate structures suitable for docking. The ionization parameter was set to Generate possible states at target pH 7.0 ± 2.0 and Epik model was used. Desalt and tautomer were checked retaining the specified chirality. 5 maximum structures were generated per ligand. Conformational search was performed for structures generated for Ligprep using MacroModel module. Structures were subjected to 5000 step conformational search. The convergence criterion was kept as 0.05 kJ/mol. The obtained structures were minimized using PRCG, Polak-Ribiere Conjugate Gradient method. The top conformations of the ligands were considered for the induced-fit docking. Conformation of the output ligands were in *S* configurations. Studies were repeated for the *R* configurations as carried out with the S configurations. The induced fit docking^26^ (IFD) was performed using Maestro, Schrödinger Suite 2021. The graphical user interface with standard protocol generates up to 20 poses. Ligands were re-docked into the structure within 30.0 kcal/mol of the best structure. XP scoring function was used in the docking stages. 5.0 Å was used for refinement and the side chains were optimized using Prime. Energy window was kept at 2.5 kcal/mol and grid was created at centroid of the workspace ligand. Further studies were also carried out by generating the grid around the binding site with top site score. The site is delineated by Ile1079-Pro1091, Thr1291- Ser 1227, Asp1244- Ile1251 and Met 1138-Pro1118. The job was run with other default settings. Minimization of the docked structure was done with the MacroModel module of Schrödinger using OPLS_2005 force field. Solvent considered for the job was water. For minimization, the PRCG (Polak-Ribiere Conjugate Gradient) method was used. This step consisted of 10,000 iterations, until the threshold convergence reached a value of 0.05 kJ/mol**.**

### In-silico ADME/T profiling of the AK778, UR778Br (S/R)

Details provided in Supplementary Material.

### Immunohistochemical analysis

Bone marrow biopsy material containing AML was randomly selected from pathology archives in a de-identified manner, and individual cases were selected based on number and abundance of diagnostic cells. Formalin fixed paraffin embedded tissue sections were cut at 4 microns and mounted on glass slides and baked for one hour at 60C to assist in tissue adhesion to the slide. Slides were then deparaffinized through xylene and graded alcohols followed by a brief rinse in wash buffer. Pretreatment of the slides was performed in a pressure cooker using a pH6 buffer for 20 min at 99C, with a brief cool down period. Slides were incubated with primary anti-IQGAP1 murine monoclonal (D3) antibody (Santa Cruz Biotechnology Inc, catalog number: sc-374307, diluation:1/100) for 60 min at room temperature. Development of the antibody was performed using the Flex HRP with DAB kit from Dako (Agilent Technologies) with hematoxylin counterstain.

### Cell viability assay

Effect of UR778Br on the viability of MOLM13, MV411, THP1 and U937 cells was determined by MTS assay. Briefly, 20,000 to 40,000 cells/well were seeded in triplicate wells of a 96 well plate and treated with indicated doses of UR778Br (0-100 µM). AK778, AB323, AS871 and AK968 were tested at concentrations ranging from 50 to 1.56 µM and incubated for 48–72 h. Cell Titre96R Aqueous One Solution Cell Proliferation Assay reagent (Promega Corp., catalog number: G3580) diluted 1:10 in complete RPMI medium was added and cells were incubated for 4 h. The optical density (OD) values were read at 490 nM wavelength using BioRad iMark microplate reader. Experiments were performed in triplicates; data are expressed as the mean of the triplicate determinations (X ± SD) of a representative experiment in % of absorbance by samples with untreated cells [= 100%].

### Colony forming unit assay

To evaluate the effect of IQGAP1 knockdown on the clonogenicity of K562, MV411 and THP1, naïve cells, the cells transfected with one of the two IQGAP1 shRNAs or scrambled shRNA were added to MethoCult H4435 Enriched (Stem Cell Technologies, catalog number: 04435) to achieve concentrations of 2000 cells/ml, 500 cells/ml and 200 cells/ml. Each condition was tested in triplicate. Colonies were counted between 7 and 21 days. To evaluate the effect of UR778Br treatment on the clonogenic potential of AML cell-lines, MOLM13, MV411, THP1, and U937 were added to MethoCult H4435 Enriched (Stem Cell Technologies, catalog number: 04435) at decreasing concentrations of 2000 cells/ml, 500 cells/ml and 200 cells/ml. UR778Br was added at indicated concentrations and DMSO was added as a control. Each condition was run in triplicate or quadruplicate.

To evaluate the effect of UR778Br on colony formation by normal bone marrow, vials of cryopreserved light density marrow aliquots were thawed in a 37 °C water bath. Nine ml of RPMI with 2% FBS were added dropwise to the cells in 1 ml of freezing media and cells were centrifuged at 1000 rpm for 5 min. Cell were then resuspended in 5 ml of RPMI. The cells were counted using a hemocytometer with viability estimated by Trypan blue dye exclusion. The cells were added to MethoCult H4435 (Stem Cell Technologies, catalog number: 04435) to achieve concentrations of 5 × 10e4 cells/ml to 1 × 10e5. UR778Br was added at indicated concentrations. The mixture was vortexed and allowed to settle. Each condition was dispersed in triplicate or quadruplicate. The plates were incubated and colonies were counted at 7-, 14- and 21 days.

To evaluate the effect of UR778Br on clonogenic potential of primary AML cells, vials of cryopreserved aliquots were thawed rapidly in a 37 °C water bath. The contents were transferred to a 15 ml conical tube, to which was added one tenth the volume of DNase (bovine pancreas deoxyribonuclease, Sigma Aldrich, catalog number: D4263-5VL). The contents were mixed gently and incubated in a 37 °C water bath for 90 s. Thereafter, 9 ml of DMEM with 2% FBS was added dropwise and mixed gently followed by centrifugation for 5 min. The supernatant was then aspirated and discarded and 70 µl of DNase was added and mixed gently. 10 ml of DMEM with 2% FBS was added slowly to resuspend followed by centrifugation for 5 to 6 min. The supernatant was discarded, and 50 ul of DNase was added and mixed gently. The pellet was resuspended in 5 ml of DMEM + 10% FBS, L-glutamine 2 mM (GIBCO) and penicillin and streptomycin (50 units and 50 mcg/ml). The cells were counted in a hemocytometer with viability estimated by Trypan blue dye exclusion. They were also added to MethoCult (Stem Cell Technologies, catalog number: 04435) to achieve final concentration between 5 × 10e4 to 2 × 10e5/ml. DMSO, UR778Br or cytarabine were added to achieve indicated concentrations. The mixture was vortexed and allowed to settle. Each condition was dispersed in quadruplicate at the volume of 0.5 ml/well of a four-well plate. The plates were incubated and colonies were counted at 7 and 14 days. Statistical analysis: The groups were compared with a one-sided Wilcoxan Rank Test.

### Cell cycle and apoptosis analyses by flow cytometry

To determine effects of UR778Br on the cell cycle and apoptosis in AML cells by flow cytometry standard protocols were followed. Briefly, harvested cells were placed in 5 mL Falcon polystyrene tubes (catalog number: 352054. Corning Incorporated. Tamaulipas. Mexico), washed twice with cold PBS pH 7.4 (catalog number: 21-040-CV. Corning. Manassas, VA. USA) and re-suspended in 1 × Annexin V binding buffer (catalog number: 51-66121E. BD Pharmingen. San Diego, CA. USA) at a cell-density of 1 × 10^6^ cells/mL followed by addition of 5 µL of Annexin V (catalog number: 51-65874X. BD Pharmingen. San Diego, CA. USA) and 5µL of propidium iodide (catalog number: 51-66211E. BD Pharmingen. San Diego, CA. USA) and incubated at room temperature for 15 min, protected from light, followed by the addition 400 µL of 1X Annexin V binding buffer. With each experiment, un-stained cells and cells stained with Annexin V-only and propidium iodine-only, were used as controls. To determine cell-cycle by flow cytometry, we used Vybrant™ DyeCycle™ Ruby Stain (catalog number: V10309. Thermo Fisher Scientific. Waltham, MA. USA) following manufacturer’s recommendations. Harvested cells were placed in 5 mL polystyrene tubes (catalog number: 352054. Falcon Corning Incorporated. Tamaulipas. Mexico) at a cell-density of 1 × 10^6^ cells/mL in cell culture media followed by the addition of Vybrant DyeCycle Ruby at a final concentration of 5 µM and incubated in the dark at 37 °C for 30 min. For co-culture experiments of AML samples with HS-5 melanoma cells, the cells were additionally stained with the HS-5 specific CD73 and CD105 human anti-CD73-BV421 (catalog number: 562430. BD Pharmingen. San Diego, CA. USA) and human anti-CD105-BV605 (catalog number: 752991. BD Pharmingen. San Diego, CA. USA). We were able to gate out any HS-5 present in the supernatant by excluding the double positive CD73 and CD105 cells. Flow cytometry experiments of stained cells were performed in the Flow Core Resource Facility, at the University of Rochester, using a Beckton Dickinson Fortessa 18-Color (Franklin Lakes, NJ. USA). Flow cytometry results were analyzed using FlowJo version 10.9.0 (Becton Dickinson & Company. Franklin Lakes, NJ. USA).

### Co-culture of primary AML with HS-5 cells

The human stromal cells HS-5 (catalog number: CL-35611 ATCC. Manassas, VA. USA), were cultured in 12 well plates (catalog number: 3513. Corning, ME. USA) with MEM alpha medium (catalog number: 12561056. Thermo Fisher Scientific. Waltham, MA. USA) and supplemented with 10% of heat-inactivated fetal bovine serum (GemCell™, catalog number: 100-500-500) at 37 °C. 80% humidity and 5% CO_2_. Cells were seeded at a concentration of 2 × 10^5^ cells/mL, 24 h prior to co-culturing with the AMLs, to allow the formation of a uniform adherent cell-layer at 4 × 10^5^ cells/well for the co-culture. On the day of the co-culture, frozen AML samples stored in liquid nitrogen, were thawed by 2 min incubation at 37ºC, transferred to 15 mL polypropylene conical tubes (catalog No. 352196. Falcon Corning Incorporated. Tamaulipas, Mexico) and resuspended in 5 mL of pre-warmed. Then, centrifuged at 1,200 rpm for 5 min at room temperature. The cells concentration was adjusted with MEM alpha medium at 4 × 10^5^ cells/mL and added to the HS-5 cell layer. Serial dilutions of UR778Br (Toronto) in MEM alpha medium were prepared to yield final concentrations of the small molecule at 25 µM, 12.5 µM, 6.25 µM, and 3.125 µM and the volume of the cell cultures were brought up to 2 mL per well with medium. Samples were incubated at 37 °C before analysis by flow cytometry in triplicate. Controls containing DMSO (catalog number: D2650. Sigma-Aldrich. Darmstadt, Germany) or media were assessed. Additional controls where both AML samples and HS-5 were incubated in the absence of each other, in the presence of UR778Br, were also included. Samples were collected at 24 and 48 h after treatment and analyzed by flow cytometry.

### Statistical analyses

The effect of UR778Br on proliferation and colony formation by cell lines was analyzed using two-sample t-tests with unequal variances on log10-transformed data. Statistical differences among the colonies formed by IQGAP1 shRNA or scrambled shRNA were analyzed by one tailed t-test using Microsoft Excel spreadsheet. The effect of UR778Br on CFU by primary AML and normal bone marrow groups were compared with a one-sided Wilcoxon Rank Sum Test.

## Results

IQGAP1 is overexpressed in primary AML cells: Rabbits (NZ white, Covance) were immunized subcutaneously weekly × 16 weeks with FDNB-modified or -unmodified human WBC cells as shown in Fig. 1A. After heat inactivation of complement, the immune sera were absorbed against pooled WBCs from normal healthy subjects. The absorbed immune sera were used to probe replicate western blots of de-identified primary human AML samples. Serum from rabbits immunized with FDNB modified WBCs recognized a different set of *antigens* than the ones by serum from rabbits immunized with unmodified WBCs (data not shown). Proteomic analyses of the affinity-purified lysates of primary AML samples were conducted using the strategy of depletion followed by enrichment as shown in Fig. 1B. In LC–MS/MS experiments done with three separate AML samples and repeated with one of those three samples, IQGAP1 was noted to be differentially recognized (Proteomics data are deposited in the PRIDE database (https://proteomecentral.proteomexchange.org/cgi/GetDataset?ID=PXD040171). Before proceeding further, western blot of the five fractions: index whole cell lysate of the AML and the fractions that did not bind to and ones that were eluted off the affinity columns sequentially were probed with a murine anti-IQGAP1 antibody (Santa Cruz Biotechnology Inc, catalog number: SC-374307) to confirm that IQGAP1 was indeed present in the immunoaffinity purified fraction. Whole cell lysate of MV411 was used as a positive control (Fig. 1C and supplementary Fig. 1). To confirm the finding we examined expression levels of IQGAP1 in nine primary AML samples by western blot. As shown in Fig. 1D, Supplementary Fig. 2, four of the nine samples showed high IQGAP1 expression, three samples showed moderate expression whereas the remaining two patient samples did not exhibit the expression of IQGAP1. Next, we compared IQGAP1 expression in AML patient samples versus normal bone marrow**.** Wright Giemsa and immunohistochemical (IHC) staining of bone marrow from AML patients (AML#1 and AML#2) showed elevated IQGAP1 protein expression (Fig. 1E) compared to marrow from normal subjects (Fig. 1F). Similarly, BloodSpot analysis^28^ showed that compared to normal hematopoietic cells, AML-subtypes including acute promyelocytic leukemia (APL) [(AML t(15:17)], acute myeloid leukemia carrying inv(16)/t(16;16), t(8;21), or 11q23 chromosomal abnormalities, and AML with complex karyotype showed increased IQGAP1 mRNA expression (*p* =  < 0.001 vs HSC, Students T test) (Fig. 1G). Kaplan–Meier analysis of the AML patients showed a trend toward worse prognosis among patients exhibiting above-median IQGAP1 expression (*p* = 0.063) (Fig. 1H).

### IQGAP1 silencing reduced proliferation and colony forming potential of leukemia cell-lines

**Figure 2:**
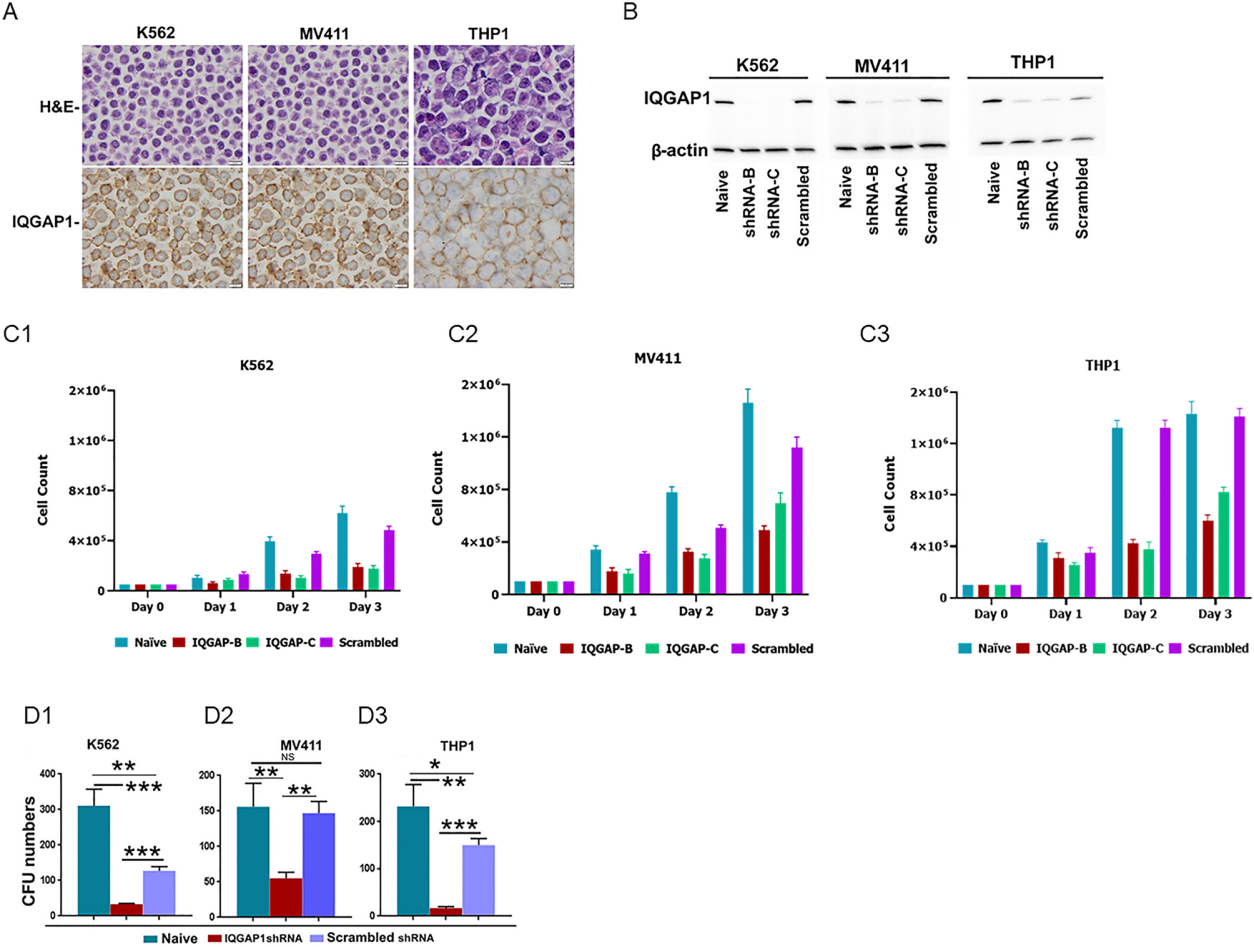
(**A**) H&E staining and IHC conducted to examine expression of IQGAP1 in K562, MV411 and THP1 cell-lines showed that IQGAP1 is highly expressed in all three cell-lines tested. 4-micron size glass slides of the formalin fixed paraffin embedded sections of cell pellets were processed and stained as described in the Supplementary Material section. (**B**–**C**) shRNA knockdown of IQGAP1 in K562, MV411 and THP1 cells, confirmed by western blot (**B**), showed reduced proliferation compared to naïve cells and cells transfected with scrambled shRNA controls. (**C**). Aliquots of naïve cells, cells exposed to IQGAP1 shRNA-B and shRNA-C and scrambled shRNA were seeded without sorting at 5 × 10e^4^ (K562) and 1 × 10e5(MV411 and THP1) cell density in 500 μL complete RPMI media in 48 well plates. Cells were counted in triplicate using a hemocytometer on days 1, 2, and 3. (**D**)**:** Clonogenicity of K562, MV411 and THP-1 cells transfected with IQGAP1-shRNA knockdown was significantly decreased compared to the naïve cells and the cells transfected with scrambled shRNA control (one-tailed T-test; **K652**, naïve vs IQGAP1shRNA-B: *p* = 0.00027, naïve vs scrambled shRNA: *p* = 0.004, scrambled vs IQGAP1 shRNA-B: *p* = 0.0023; **MV411**, naïve vs IQGAP1shRNA-B: *p* = 0.0018, naïve vs scrambled shRNA: *p* = 0.68, scrambled vs IQGAP1 shRNA-B: *p* = 0.0025; **THP-1**, naïve vs IQGAP1shRNA-B: *p* = 0.0014, naïve vs scrambled shRNA: *p* = 0.0125, scrambled vs IQGAP1 shRNA-B: *p* = 0.0007)..

H&E and IHC staining were performed on K562, MV411 and THP1 cell lines to confirm IQGAP1 expression (Fig. 2A) prior to shRNA knockdown. Two different shRNAs (sequence details provided in Materials and Methods section) were used to knock down the expression of IQGAP1 in the K652, MV411 and THP1 cell lines. The knockdown of IQGAP1 was confirmed by probing whole cell lysates of naïve cells, cells transfected with the two shRNAs and scrambled shRNA with murine anti-IQGAP1 monoclonal antibody (Fig. 2B and Supplementary Figs. 3 and 4). Compared to naive and scrambled shRNA transfected K652, MV411 and THP1 leukemia cells, transfection with IQGAP1 shRNAs caused robust suppression of proliferation (Fig. 2C). Similarly, shRNA knockdown of IQGAP1 in K652, MV411 and THP1 leukemia cells resulted in the significant reduction in the number of colonies formed compared to those generated from cells transfected with scrambled shRNA or untransfected controls. Data from one of three experiments are shown in Fig. 2D

### Virtual screening identified small molecule inhibitors of IQGAP1

**Figure 3:**
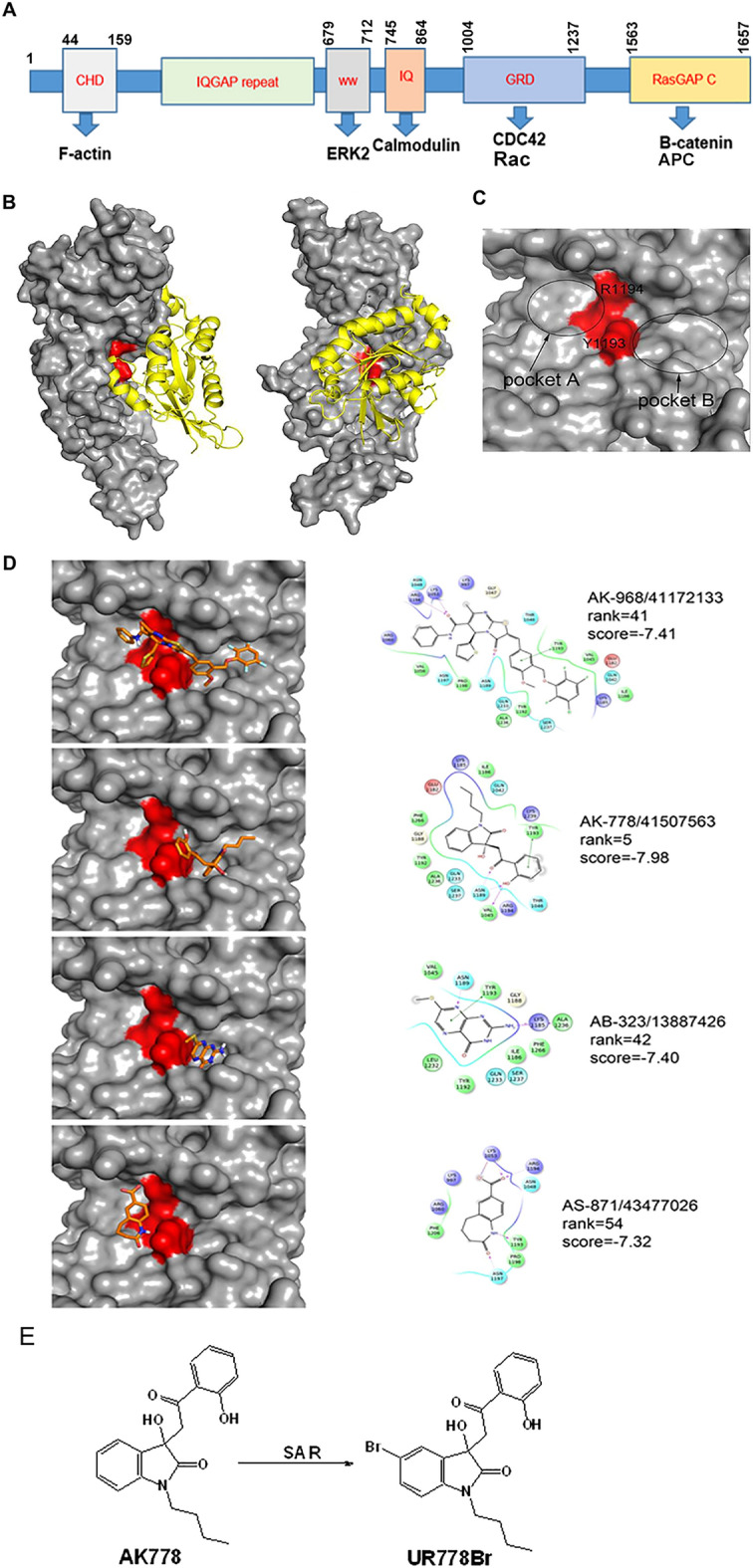
(**A**): Schematic diagram of the structural subunits of IQGAP1 protein. Blue arrows indicate listed proteins. (**B**): Crystal structures of IQGAP1 GRD-domain used for the *in-silico* screening of the compound library are shown. (**C**): Binding pockets into which the library of compounds was docked are shown. (**D**-left): snapshots of the hits docked with IQGAP1-GRD domain are shown. (**D**-right): Chemical structures of the hits, ranks and binding scores are shown. The highest ranked hit compound AK-778 was chosen for further studies. (**E**): Schema of the structure–activity relationship (SAR) starting from AK778 structure (left) leading to the identification of UR778Br (right) is shown.

We conducted a virtual screening of 212,966 compounds (described in Supplementary Material) to identify a set of hits against IQGAP1 protein (Fig. 3A). Binding pockets utilized for docking are shown (Fig. 3B–C). Four structurally different classes of the small molecule hits ranked 5-54 based on their binding energy scores were identified (Fig. 3D). Hits AK778 (rank 5), AB323 (rank 42) and AS871 (rank 54) were tested for their effects on the proliferation of leukemia cells (data not shown). Highest ranked hit (AK778) showed the most potent inhibition of leukemia cells. To understand the structure–activity relationship, a set of ten close structural analogs of AK778 were identified (Profacgen, Shirley, NY). Screening revealed UR778Br as the most potent structural analog of AK778. From the limited but focused structure–activity relationship studies, it emerged that the installation of a bromine atom on the 7th carbon of the indole ring enhanced the anti-proliferative activity of AK778 (Fig. 3E). To further probe the structure–activity relationships, additional analogs of UR778Br were synthesized keeping the 7-bromine substitution intact but with rotating the –OH group. Alterations in the –OH position led to the loss of activity suggesting that the hydroxyl group on the ortho-position in the phenyl ring is essential (Supplementary Fig. 6).

### In silico docking reveals binding sites of UR778Br in IQGAP1

**Figure 4:**
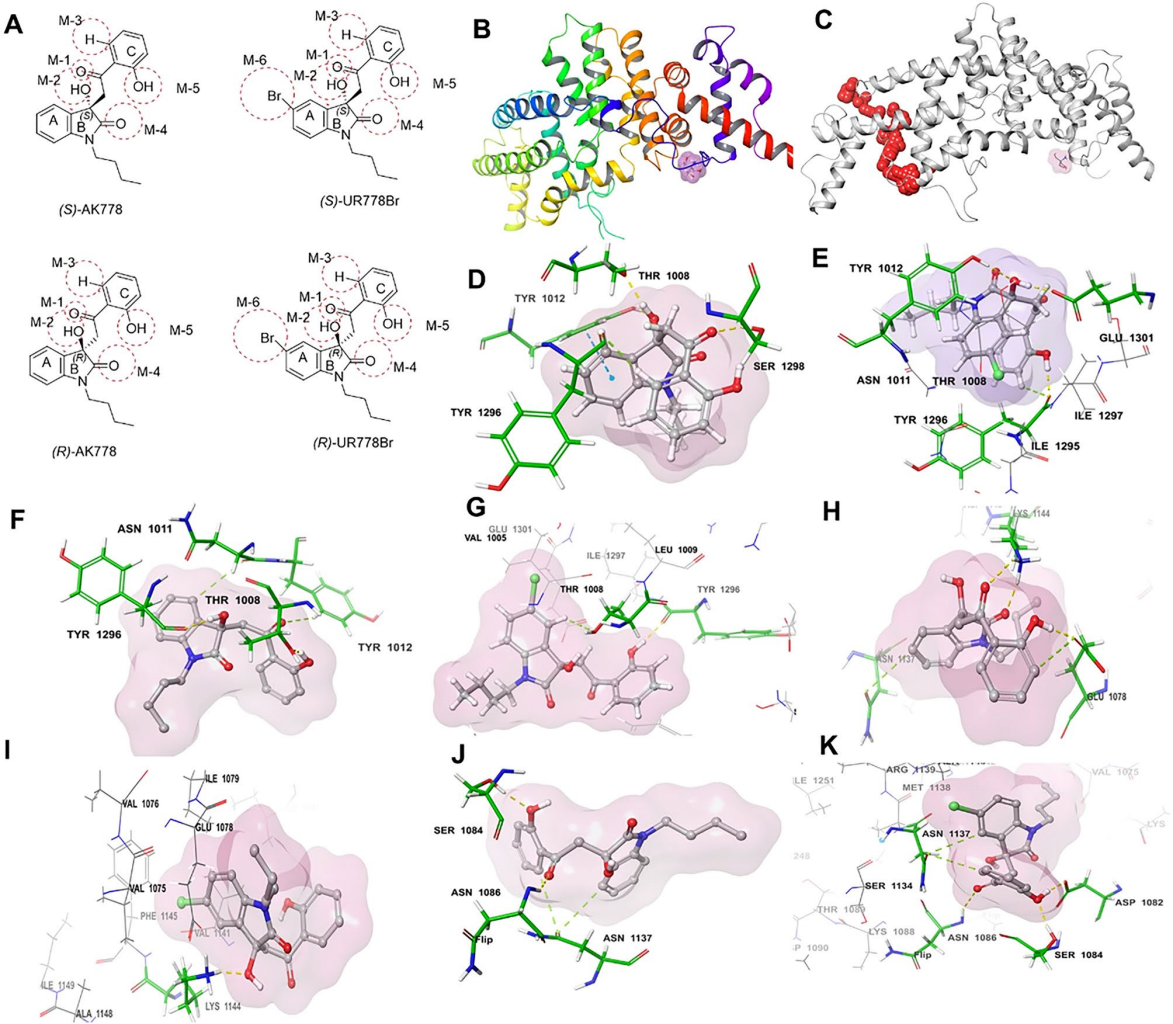
(**A**): Chemical structure of *R* and *S* configurations of AK778 and its analog, UR778Br with binding motifs (M) highlighted with a red dotted circle. Motifs assignments were observed to interact with the residues. (**B**): Crystal Structure of Ras GTPase-activating-like protein IQGAP1 with TRS, Tris buffer. The crystal structure is represented in ribbon form in which the unique ligand TRS (Tris buffer) is highlighted with purple surface in the loop region. (PDB ID: 3FAY). (**C**) Location of the binding site with top site score contrary to the active site specified by TRS. (**D**–**E**) Binding modes of *(S)*-AK778 and *(S)*-UR778Br and with the residues at the binding site identified around TRS. (**F**–**G**): Binding modes of *(R)-*AK778 and *(R)-*UR778Br with the residues at the binding site identified around TRS. (**H**–**I**): Binding modes of *(S)*-AK778 and *(S)*-UR778Br and with the residues at the top ranked site*.* (**J**–**K**): Binding modes of *(R)*-AK778 and *(R)*-UR778Br and with the residues at the top ranked site. Representation of the binding interactions: п-п stacking (blue dotted lines), H-bond (yellow dotted lines and aromatic H-bonds (green dotted lines).

The ligands AK778 and its analog UR778Br, along with their respective R and S configurations (binding motifs: Fig. 4A), were examined for binding affinities at the site around TRS (Fig. 4B) and the top-ranked site (Fig. 4C), SiteScore: 0.95 and Dscore: 0.87, identified by SiteMap analysis. At the site around TRS, as shown in Fig. 4D, ring A of *(S)*-AK778 interacted with Tyr 1012 via п-п stacking (blue dotted lines) at a distance of 5.41 Å. The Hydroxyl group (M-2) formed a hydrogen bond with Thr1008 and Tyr1012 (yellow dotted lines). Hydrogen (M-3) interacted with Tyr1296 via an aromatic hydrogen bond (green dotted lines). The carbonyl motif (M-4) interacted with Ser1298 via hydrogen bonding. Tyrosine and isoleucine are hydrophobic, whereas threonine and serine have polar uncharged side chains. (S)-AK778 formed 12 hydrophobic contacts in the docked complex with Thr1008, Tyr1296 and Tyr1012 (Supplementary Table 1B). As shown in Fig. 4E, M-2 of (S)-UR778Br interacted with Glu1301 and Tyr1012 (yellow dotted lines) via hydrogen bonding, unlike M-2 of AK778. The bromine atom was surrounded by hydrophobic (Tyr1296, Ile1295, Ile1297) and polar (Asn1011, Thr1008) residues. Hydrophobic interactions are essential to determine a ligand's stability at the receptor's binding site. (S)-UR778Br formed 22 hydrophobic contacts (Supplementary Table 1B). The five carbon atoms of the phenyl group (Ring A) of the indole ring form hydrophobic contacts with Tyr1296, Thr1008, and Tyr 1012. The bromine atom (M-6) of (S)-UR778Br formed hydrophobic contacts with Tyr1296, Ile1295, Ile1297 and Thr1008. Unlike the (S) configuration, (R)-AK778 (Fig. 4F) did not interact via п-п stacking. The carbonyl group (M-1) linking the indole moiety and ring C did not form H-bond, unlike (S)-AK778. The carbonyl group (M-1) forms an aromatic H-bond with Tyr1012. The hydrophobic region of (R)-AK778 consists of Tyr1012 and Tyr1296. Unlike (S)-AK778, it reacts with a hydrophilic residue, Asn1011. In (R)-UR778Br (Fig. 4G), the hydroxyl group (M-5) of the phenyl ring C forms H-bond with Tyr1296. In the indole ring, a hydrogen atom on the carbon adjacent to the carbon attached to bromine forms an aromatic H-bond with Thr1008. It forms hydrophobic contact with Tyr1012, Thr1008, Glu1301, and Lys1000. The bromine moiety (M-6) interacts with Ile1297, Glu1301, Asn1304, and Val1005 via van der Waals interaction. At the top-ranked site identified by SiteMap analysis, In Fig. 4H, *(S)-*AK778 formed three H-bonds with Lys1144 and Glu1078. The linker carbonyl group (M-1) forms one H-bond with Lys1144. It also formed two aromatic H-bond s with Asn1137 and Glu1078 (M-5). M-2 of (S)-UR778Br interacts with Lys1144 via one H-bond in Fig. 4I. In Fig. 4J, *(R)-*AK778 formed two H-bonds with Ser1084 (M-5) and Asn1086 (M-1). M-3 of ring C and one of the hydrogen atoms of ring A form two aromatic H-bonds with Asn1137. *(R)-*UR778Br (Fig. 4K) interacted with Asn1086 (M-1), Ser1084 (M-5), and Asp1082 (M-5) via two aromatic H-bonds and one H-bond, respectively. Both of these ligands interacted with Asn1137, Asn1086, and Ser1084. An MDS video is presented as Supplementary Video-1 to describe the binding of UR778Br with IQGAP1-GRD domain residues. Overall, as described in Supplementary Table-1 (A:D), energy calculations and Glide gscore (docking score) showed that UR778Br was more stable than precursor AK778, notably (S)-UR778Br was more favored. Specifically, the docked complex with (S)-UR778Br forming 40 hydrophobic contacts at the top-ranked site was most favorable. Amongst all the configurations of the ligands, (S)-UR778Br was observed to be the best ligand to bind to the protein (PDB ID: 3FAY) at the site identified by SiteMap analysis. Complete energy calculations are shown in Supplementary Table-1 (A-D).

### In silico ADME analysis of AK778 and analogs

The *in-silico* prediction of the ADME/T properties favored the compound UR778Br. Therefore, we hypothesize that UR778Br, analog of AK778, may show better biological activity at the newly identified active site which may be validated by further experimental studies. UR778Br analog has greater polar surface area due to the presence of bromine compared to AK778. It is more lipophilic due to C–Br bond; this justifies the value for log S. Negative values of log S values of UR778Br was greater than AK778. This implies that, UR778Br has lower value of aqueous solubility than AK778. The values of log S and logPo/w are inversely proportional to each other. None of the ligands violate Lipinski Rule of Five and Jorgensen Rule of three. All the four ligands are drug like and orally available. Molecular weight, lipophilicity, solubility and flexibility of the ligands determine their penetration. The four candidates have 100% oral absorption in the gastrointestinal tract. The blood/brain partition coefficient is used to predict accessibility of a drug candidate to the central nervous system. UR778Br is comparatively less polar than AK778 and will cross blood–brain barrier; greater negative values suggest the polarity and the inability to cross the blood–brain barrier. UR778Br is more active towards central nervous system. Positive values of logPo/w suggest lipophilicity of a drug candidate. Lipophilic drug candidates are distributed in the lipid bilayers whereas polar drug candidates get distributed in the blood serum (Supplementary Table-2).

### UR778Br reduced proliferation, caused G2M cell cycle arrest, induced apoptosis and suppressed colony forming potential in leukemia cell lines

**Figure 5:**
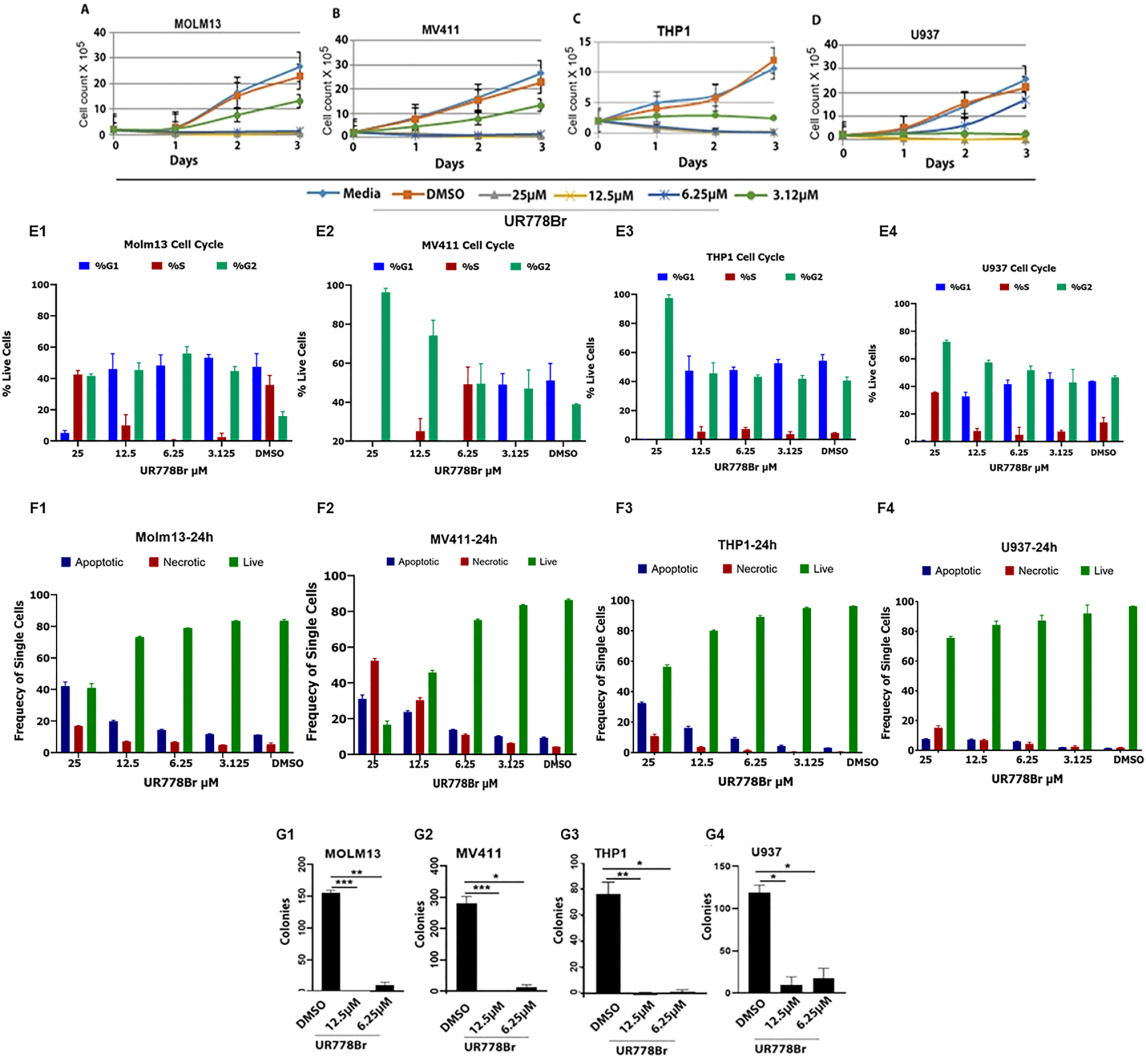
(**A**–**D**). MOLM13, MV411, THP1 and U937 cells (2 × 10^5^ cells/ml) treated with indicated concentrations of UR778Br compared to DMSO showed reduced cell counts during the 3 days of treatment. Cells from three independent wells per condition were counted on days 1–3. (MOLM13: UR778Br 3.125 μM vs DMSO at day 3, *p* = 0.02; MV411: *p* = 0.008, THP1: *p* = 0 0001, and U937 at 6.25 μM: *p* = 0.08 and at 12.5 < 0.0001). (**E1-4**): Exposure of the four cell lines to a range of doses of UR778Br compared to DMSO fir 24 h caused G2M cell cycle arrest and (F1-4) induced apoptosis. (G1-4) MOLM13, MV411, THP1 and U937 cells seeded at 2000 cells/ml density in triplicate were added to MethoCult H4435 Enriched (Stem Cell Technologies, Catalog number: 04435) and UR778Br was added to achieve indicated concentrations. DMSO was added as a control. Colonies in three or four replicated wells were counted between 7 and 14 days. (*p* value 6.25 μM UR778Br vs DMSO were for: MOLM13: *p* = 0.006, MV411: *p* = 0.02, THP1: *p* = 0.01, and U937: *p* = 0.03).

Exposure of MOLM13, MV411, THP1 and U937 to a range of doses of UR778Br compared to DMSO reduced proliferation over 72 h in a dose dependent manner (Fig. 5A–D). UR778Br also caused G2M cell cycle arrest (Fig. 5 E1–4) and induced apoptosis at 24 h (Fig. 5 F1–4). The Effect of UR778Br on MOLM13, MV411, THP1 and U937 colony forming potential was measured by counting colony-forming units in MethoCult Enriched medium in 14 days. The number of colonies formed by cells treated with UR778Br 6.25 µM were significantly lower than those exposed to DMSO (Fig. 5 G1–4).

### UR778Br dose-dependently decreased viability, caused apoptosis and decreased colony formation potential in primary AML cells while sparing normal bone marrow cells

**Figure 6:**
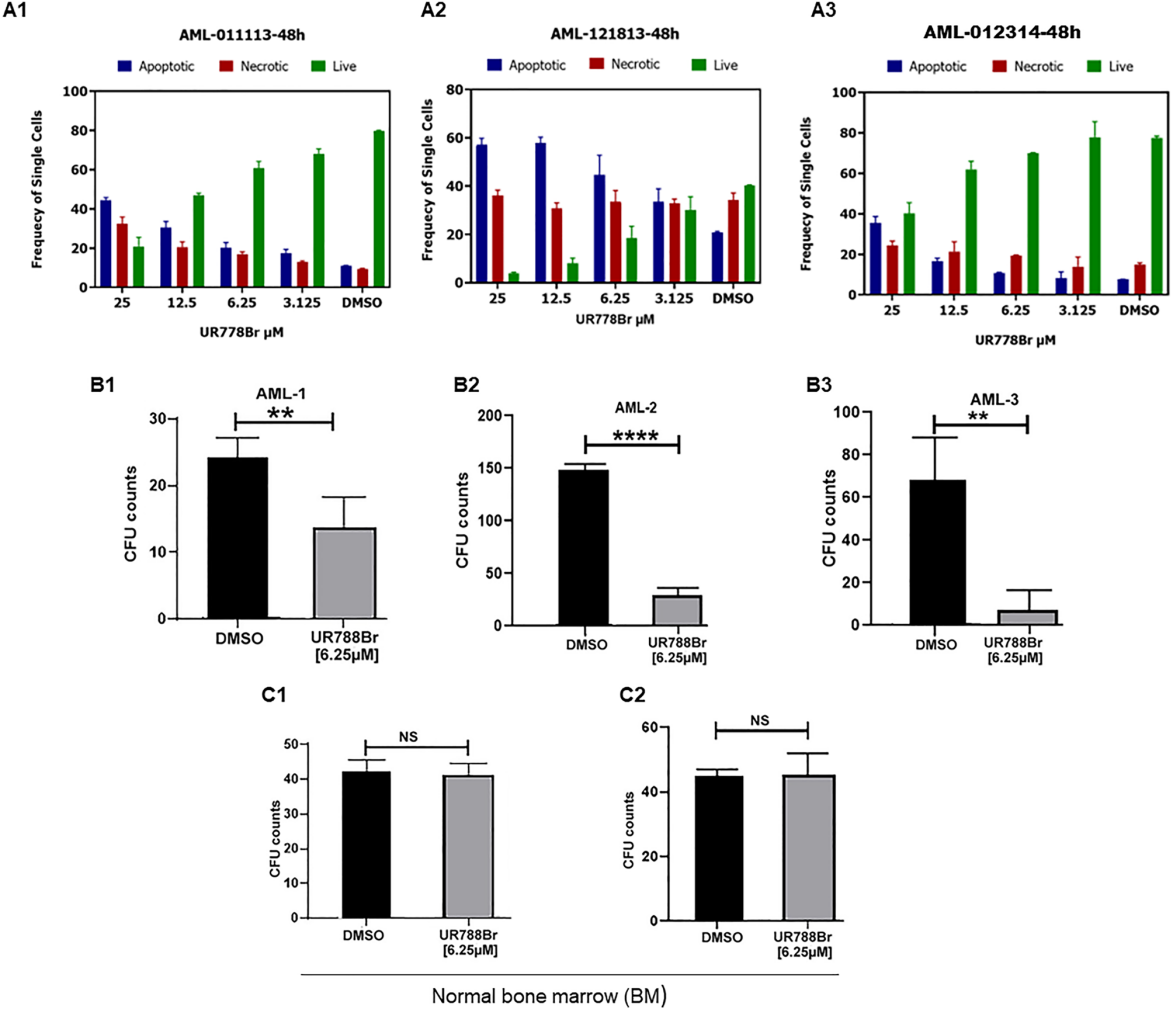
(**A1–3**) Deidentified AML cells were cocultured with HS-5 melanoma cells and exposed to a range of concentrations of UR778Br and DMSO for 48 h, the cells were additionally stained with human anti-CD73-BV421) and human anti-CD105-BV605) to gate out any HS-5 present in the supernatant by excluding the double positive CD73 and CD105 cells. The data show dose dependent induction of apoptosis. (**B 1–3**): UR778Br (6.25 μM) reduced the colonies formed by the de-identified primary AML cells (unpaired two-tailed t-test; AML-1: *p* = 0.0085; AML-2: *p* =  < 0.0001, AML-3: *p* = 0.0014). 50,000 to 100,000 cells/ml primary AML cells were dispersed in quadruplicate wells in MethoCult H4435 Enriched medium. UR778Br was added to achieve 6.25 μM concentrations. DMSO was added as a control. Colonies were counted on the day 15**.** (**C1–3**): UR778Br did not affect the clonogenicity of two normal bone marrow samples (one-sided Wilcoxon Rank Sum Test *p* = 0.2533 (**C1**) and 0.3313 (**C2**)). Normal bone marrow cells were thawed and dispersed at 50,000 to 100,000 cells/ml in quadruplicate suspended in MethoCult H4435 Enriched medium. UR778Br was added to achieve 6.25 μM concentration. DMSO was added as control. Colonies were counted on the day 14.

Primary AML cells co-cultured with HS-5 were exposed to DMSO and a range of concentrations of UR778Br for 48 h. Analysis by flow cytometry revealed dose-dependent induction of apoptosis (Fig. 6A1–3). Notably, HS-5 populations and viability were not affected. (Supplementary Table 3). When examining the effects of UR778Br on colony formation of AML cells, we observed that compared to DMSO, exposure to UR778Br (6.25 µM) caused significant decrease in the number of colonies formed by primary AML cells in MethoCult Enriched medium (Fig. 6B1–3. Example of an AML CFU, supplementary Fig. 5). In contrast to primary AML samples, treatment with UR778Br at 6.25 µM did not decrease clonogenicity of two normal bone marrow samples (Fig. 6C1–2) (one-sided Wilcoxon Rank Sum Test p = 0.2533 and 0.3313 in (Fig. 6C1–2), respectively).

## Discussion

More than 21,000 new cases and over 11,400 deaths from AML were predicted in the US in the year 2022^9^. Continuous-infusion of cytarabine and an anthracyclines has been the mainstay of induction chemotherapy. After remission induction, allogeneic hematopoietic-cell transplantation, when feasible, can result in 35–40% long-term survival in patients younger than 60 years of age. However, prognosis of older patients who are unable to receive intensive chemotherapy remains dismal, with median survival of 5–10 months^1^. Analysis of the SEER database revealed that during the 3 decades from 1977 to 2006, the overall relative survival had not improved, especially among patients aged ≥ 75 years^10^.

In recent years drugs targeting alterations in FLT3, BCL2, IDH1, Hedgehog pathway have been tested alone or in combination with hypomethylating agents as well as conventional chemotherapy in subsets of AML^28^. Combination of azacitidine and venetoclax has shown a modest increase in the survival^10^ in patients with median age of 76. However, age matched patients treated with the combination off-trial had inferior outcomes^30^. Combination of midostaurin plus chemotherapy was superior to chemotherapy in patients with previously untreated AML with FLT3 mutation in patients between the ages of 18–59^31^. Gilteratinib was superior to salvage chemotherapy in relapsed or refractomy FLT3-mutated AML. However, the median age of patients treated was 62^32^. In contrast the median age of AML patients at diagnosis is about 68 years. Clearly, there is an unmet need to identify and validate driver-like therapeutic targets and to develop pharmacologic modulators of those targets to improve outcomes in AML, particularly in older patients.

This study identified IQGAP1 as being significantly overexpressed in various AML subtypes compared to normal bone marrow. Genetic perturbation (knockdown) validated dependency of AML on IQGAP1 manifested by reduced survival and colony formation (Fig. 2D) providing the proof of concept that IQGAP1 may be a therapeutic target in AML. Acting as a scaffolding protein, IQGAP1 participates in various biological activities, including signal transduction, cytoskeletal dynamics, cytokinesis, cell polarizations, cadherin mediated cell–cell adhesion, cell polarization and actin reorganization to promote cell migration^33^, invasion, and cell proliferation, many of which are involved in tumorigenesis. In addition to AML as reported here, IQGAP1 is also overexpressed in breast-, colon-, pancreatic, gastric-, lung-, ovarian- and liver cancers^14,15^. Furthermore, overexpression of IQGAP1 is associated with worse prognosis in breast^34,35^, head&neck^36^, pancreas^37^, liver^38^, colorectal^39^, gastric^40^, lung^41^ and ovarian cancers^42^. Similar to AML, as reported here, disrupting IQGAP1-mediated PI3K expression and functions via IQ3 peptide inhibited human HNSCC cell survival, proliferation, migration, and invasion, indicating importance of IQGAP1 as a therapeutic target in a broad range of malignancies^43,44^. Importantly, IQGAP1 appears to not serve critical non-redundant functions. Li et al. had shown that IQGAP1—^*/—*^was not embryologically fatal; and null mutant pups arose at normal frequency, showed no defects during most of their adult life except late onset gastric mucosal hyperplasia relative to wild type mice. Therefore, targeting IGAP1 can emerge as a safer therapeutic option than chemotherapies which affect proliferating cells indiscriminately^45^.

At the time, when we undertook the described experiments, a small molecule targeting IQGAP1 had not been described in the literature. However, two published reports indicated that pharmacologically targeting IQGAP1 was feasible and effective. A small molecule inhibitor of Rac1/Cdc42 GTPases, which are upstream of IQGAP1, was reported to suppress growth of primary human prostate cancer xenografts^46^ and a 40 amino acid ERK1/2-binding IQGAP1 WW domain peptide that disrupted IQGAP1-ERK1/2 interactions was shown to inhibit RAS- and RAF-driven tumorigenesis^47^. Therefore, we conducted virtual screening of a library of small molecules to identify candidate small molecules targeting the GRD domain of IQGAP1. We chose the GRD domain for in silico screening because its crystal structure had been described and because Cdc2/RAC1 bind to the GRD domain and signaling via that axis is described to be involved in leukemogenesis as well as in cell cycle progression, survival, invasion and migration, each of which are characteristics of malignancies^16,17^. Recently, Sayedyahossein et al^48^ reported identification of small molecules that disrupt binding of Cdc42 and IQGAP1 and inhibit proliferation and migration of breast carcinoma cells lending further credence to the approach of targeting the GRD domain of IQGAP1.

We conducted virtual screening of 212,998 small molecules to identify a set of tractable small molecules (hits) that were amenable to optimization via structure activity relationship guided medicinal chemistry to generate the inhibitors of targeting the GRD domain of IQGAP1. To screen the library of compounds, structures of GRD and CDC42 onto GAP-334 and Ras were superimposed, respectively to arrive at a binding model of GRD with CDC42 (Fig. 3B) which was consistent with published literature based on the T1046 positioning near the GDP from CDC42^21^. The region around ^1192^YYR^1194^ was chosen as the binding site for virtual screening. Of the four predicted ‘hits’ by the virtual screening campaign a focused set of 10 structural derivatives of the best hit AK778 were further rescreened to select UR778Br, which showed most potent anti-proliferative activities. UR778Br has a bromine installed on the indole ring additionally. UR778Br demonstrated desirable anti-leukemic effects: decreased viability, proliferation and caused dose-dependent G2/M arrest in AML cell lines. In addition, UR778Br also inhibited the viability and colony formation of human primary AML cells without affecting normal bone marrow cells, which suggests that targeting IQGAP1 may result in lower hematologic toxicities than cytotoxic chemotherapy. Molecular docking studies indicate that S-isomer interacts differently than the R-isomers. While both R and S-isomer interact with Tyr1296, S-isomer interacts with Tyr1012 and Glu1301 picking up more hydrophobic interactions than R-isomer which interacts with Thr10008. S-isomer binds better than the R-isomer (Glides score: -5.27 vs -4.84), has superior potential and electrostatic energy than R-isomer which scores better with Van der Waal energy (Fig. 4K). UR778Br carries desirable drug-like characteristics^27^. Calculated log P(3.28), cLogP (4.84) and tPSA (total polar surface area: 77.84) along with a molecular weight of 417 suggest that UR778Br carries desirable druggability features^27^Supplementary Table 2. We resolved both isomers of UR778Br using chiral HPLC in the anticipation that one of the enantiomers will exhibit superior activity. However, both isomers showed similar effect on leukemia cell-lines’ proliferation as the racemic mixture. Future studies will focus on optimization of the structure of UR778Br followed by in vivo evaluation in xenografts of IQGAP1 expressing human AML cell lines as well as on engraftment and survival of primary AML samples in NOD.Cg-Prkdc^scid^Il2rg^tm^^1^Wjl (NSG) mice.

In summary, the data presented show that a significant percentage of primary AML samples overexpress IQGAP1 and that a small molecule designed to target the GRD domain of IQGAP1 has the potential to inhibit AML with a favorable therapeutic index.

### Supplementary Information

Supplementary Information.

## Data Availability

The proteomics data generated and analysed during this study are deposited in the publicly accessible PRIDE database, accession number PXD040171. (https://proteomecentral.proteomexchange.org/cgi/GetDataset?ID=PXD040171).
