# Supplementary material for: In silico predicted compound targeting the IQGAP1-GRD domain selectively inhibits growth of human acute myeloid leukemia
Source: Sci Rep. 2024 Jun 4;14:12868. doi: 10.1038/s41598-024-63392-2 (PMC11150481; doi:10.1038/s41598-024-63392-2)
Supplement: Supplementary file 1 — Supplementary Information. [file 41598_2024_63392_MOESM1_ESM.docx]

***In silico* predicted compound targeting the IQGAP1-GRD domain selectively inhibits growth of human acute myeloid leukemia**

Deepak M. Sahasrabudhe*^1,2^, Jane L. Liesveld^1,2^, Mohammad Minhajuddin^3^, Niloy A Singh^2^, Subhangi Nath^4^, Vishuwes Muthu Kumar^4^, Marlene Balys^5^, Andrew G Evans^6^, Mitra Azadniv^2^, Jeanne N Hansen^7^, Michael W Becker^1,2^, Ashoke Sharon^4^, V Kaye Thomas^8^, Richard G Moore^9^, Manoj K Khera^10^, Craig T Jordan^3^, Rakesh K Singh*^9^.

**Supplementary Material:**

**Addendum to the Background**: We summarize the previously published data as background information that provided the impetus to conducting the experiments and results reported in this manuscript. Disclosure: These previously published experiments were done in the laboratory of the corresponding author’s (DMS) late father. The data reported in this manuscript are distinct and stand on their own merit.

*Observations*: Two observations formed the basis of a testable hypothesis. First, there was a reciprocal relationship between the electrical charge of antigens and the antibodies it elicited^1^, and second, myeloid as well as lymphocytic leukemic cells were more electronegative compared to their normal counterparts^2^. The *hypothesis* was that antibodies raised against normal WBCs that have been rendered more electronegative would cross-react with leukemic cells.

*In vitro modification of WBCs*: Normal human lymphocytes are estimated to contain 9 x 10^5^ positively charged amino terminal groups^3^. Since the net surface charge is the algebraic sum of positive and negative charges on the cell surface, it was predicted that progressive *in situ* modification by chemical manipulation of positively charged amino terminal groups would result in net increase cell surface negative charge. Fluoro 2,4 dinitrobenzene (FDNB) is highly reactive and combines with amino groups at physiologic pH and at room temperature^4^. It was empirically determined that incubation of 1 x 10 ^(7)^ WBCs with 31 picograms of fluorodinitrobenzene (FDNB) which, based on Avogadro’s number, corresponds to 10^11^ molecules or 10^4^ molecules per cell, rendered the WBCs electronegative to the same degree as leukemic cells^5^.

*Cross Reactivity of Immune Sera*: Rabbits were immunized with peripheral blood WBCs that had been modified by incubation with FDNB. The immune sera were extensively absorbed with WBCs from normal healthy donors. The absorbed sera selectively agglutinated CML, CLL, AML, and ALL cells from patients^6^.

*Reproducibility*: In addition to rabbits, immunization of goats and a horse with FDNB modified WBCs elicited an antibody response that cross-reacted against a broad range of leukemias, as determined by agglutination and Ouchterlony gel diffusion^7^. No toxicity was observed in the animals.

*Immunoaffinity enrichment*: The antigenic moiety from FDNB-modified WBCs was purified using an immuno-affinity column. Immunization of mice with the eluted fractions elicited an antibody response that cross-reacted against human leukemias^8^.

In summary, immunization of rabbits with human peripheral blood WBCs that had been incubated with FDNB elicited high titer antibodies that agglutinated a broad spectrum of human leukemia cells in repeated experiments. Similar high-titer antibodies were elicited in goats and a horse indicating that *this was a reproducible and robust phenomenon.* However, given the imitations of technology in the 1970’s the affinity purified antigenic moiety could not be characterized.

We sought to determine the molecular nature of the antigenic moiety. The results are reported in the manuscript.

**IQGAP1 IHC methods (Figure 2 A)**

Formalin fixed paraffin embedded tissue sections were cut at 4 microns and mounted on glass slides and baked for one hour at 60C to assist in tissue adhesion to the slide. Slides were then deparaffinized through xylene and graded alcohols followed by a brief rinse in wash buffer. Pretreatment of the slides was performed  in a pressure cooker using a pH6 buffer for 20 minutes at 99C, with a brief cool down period.  Slides were incubated with primary anti-IQGAP1 murine monoclonal (D3): (Santa Cruz Biotechnology Inc. catalog number: sc-374307diluted to 1:100 for 60minutes at room temperature.   Development of the antibody was performed using the Flex HRP with DAB kit from Dako (Agilent Technologies) and hematoxylin counterstain.

**In-silico ADME/T profiling of the AK778, UR778Br (S/R):** The ligands were evaluated for “drug likeness” and “oral availability” on the basis of Lipinski’s Rule of Five and Jorgensen Rule of Three. Polar surface area, octanol-water partition coefficient, aqueous solubility, central nervous system activity, blood-brain barrier and percentage human oral absorption were predicted. These ADME/T properties were calculated using QikProp program. The acceptable ranges of the properties are as follows: MW: 130-725g/mol, PSA:7.0 – 200.0, Predicted octanol/water partition coefficient (logP_o/w_): 2.0-6.5, Lipinski rule of five: MW < 500, QPlogP_o/w_ < 5, H-bond donors ≤ 5, H-bond acceptor ≤ 10, Jorgensen rule of three: QPlogS > -5.7, QPPCaco> 22 nm/s, Primary Metabolites < 7, CNS: -2 (inactive)-+2 (active), logBB: Predicted brain/blood partition coefficient (logBB): 3.0-1.2, Percent human oral absorption: >80%(high), <25%(poor). Positive values of logP_o/w_ suggest lipophilicity of a drug candidate. Lipophilic drug candidates are distributed in the lipid bilayers whereas polar drug candidates get distributed in the blood serum*.*


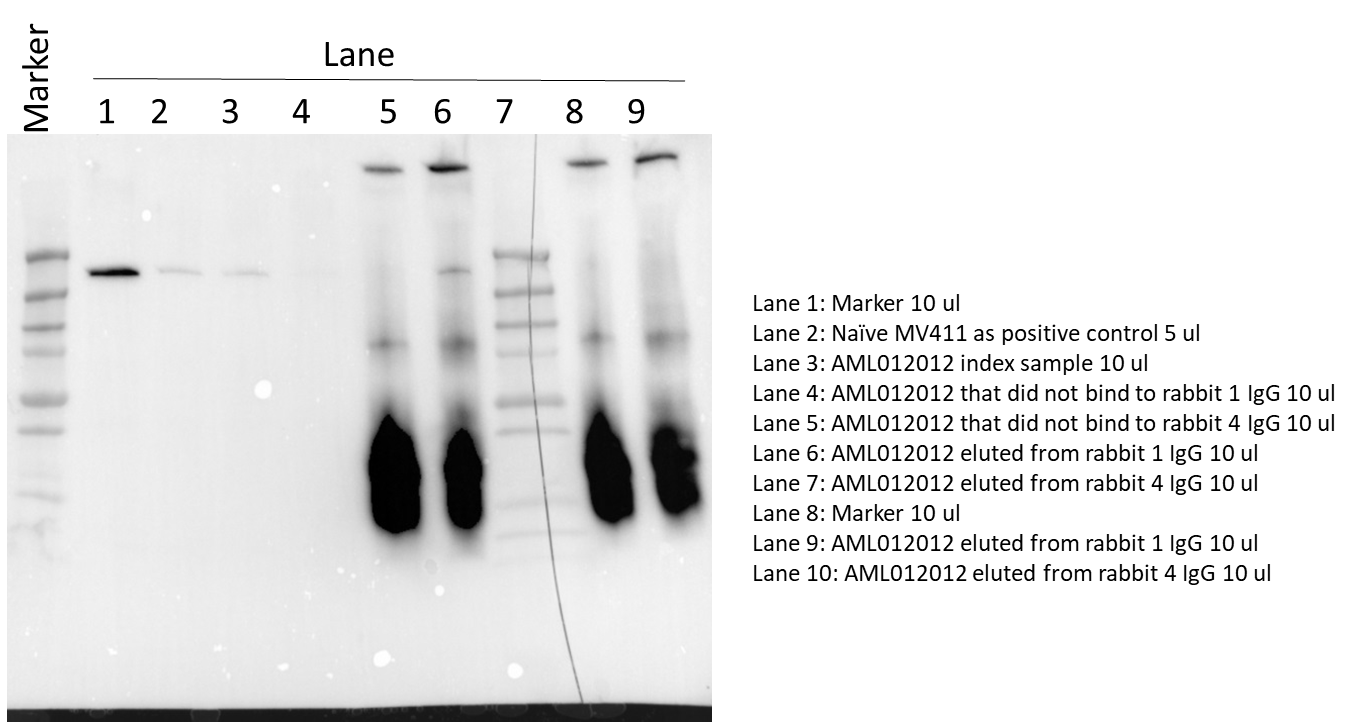


**Supplementary Figure-1:** Western blot of the five fractions: index whole cell lysate of the AML and the fractions that did not bind to and ones that were eluted off the affinity columns sequentially were probed with a murine anti-IQGAP1 antibody (Santa Cruz Biotechnology Inc, cat#SC-374307) to confirm that IQGAP1 was indeed present in the immunoaffinity purified fraction. Whole cell lysate of MV411 was used as a positive control.


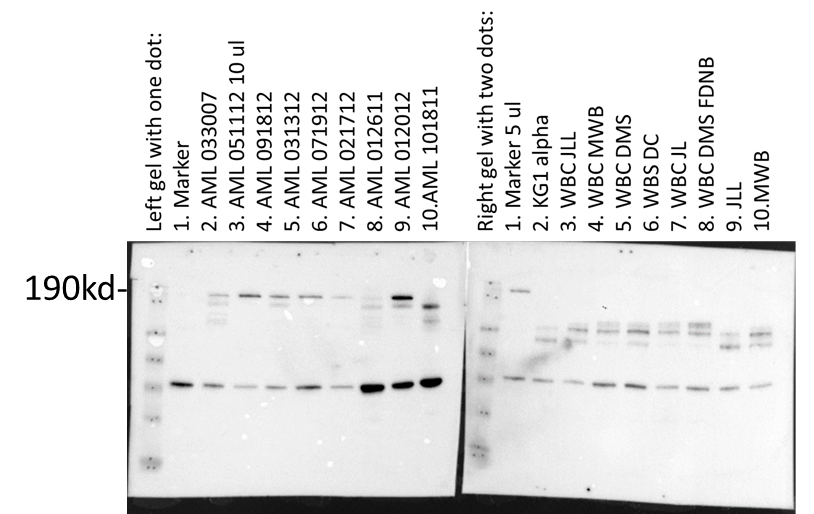


**Supplementary Figure-2:** Unaltered scan of western blot membrane depicting expression of IQGAP1 in primary AML cells isolated from patients. Conditions are described on the top of wells.

**
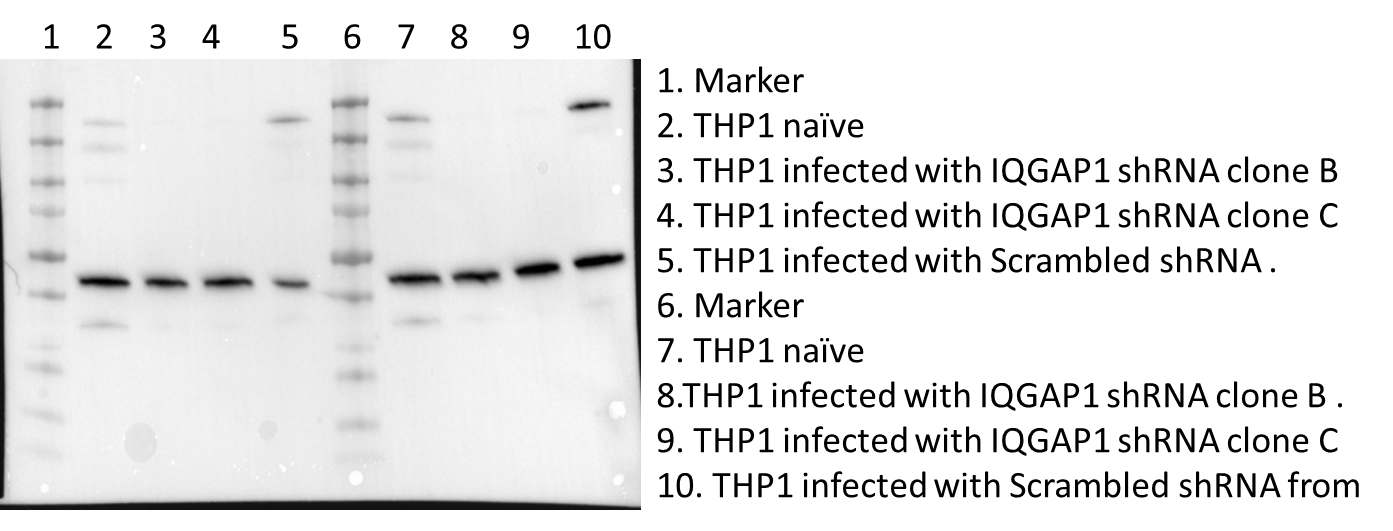
**

**Supplementary Figure-3:** Unaltered scan of western blot membrane of shRNA knockdown of THP1 cells compared to scrambled shRNA oligo. Conditions are described on the right hand side.


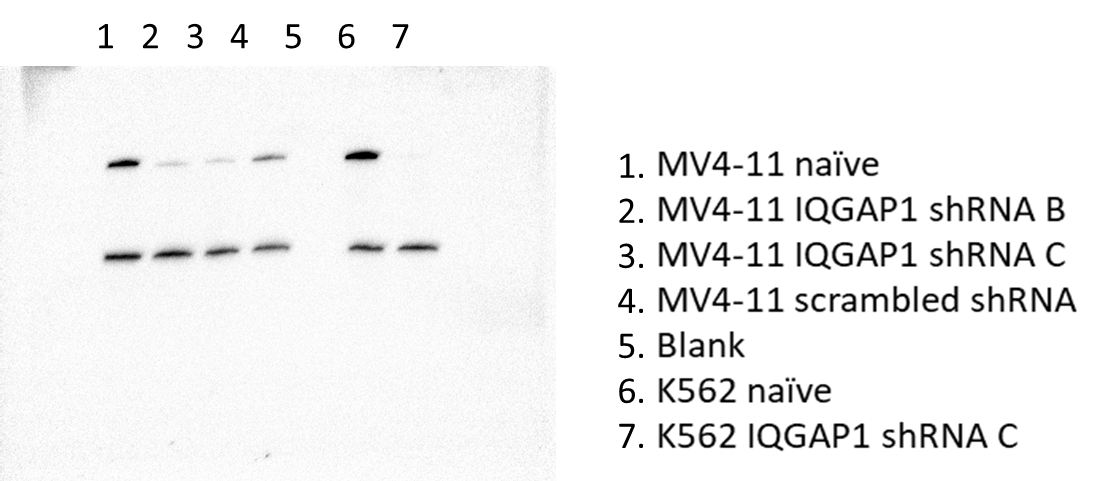


**Supplementary Figure-4:** Unaltered scan of western blot membrane of shRNA knockdown of MV4-11 and K562 cells compared to scrambled shRNA oligo. Conditions are described on the right hand side.

**Supplementary Video-1:** Molecular simulation was used to model the 3-D structure of a binding site on IQGAP1. Once identified, the binding site is categorized based upon hydrogen bonding or electrostatic or hydrophobic interactions. The interaction sites were used to limit the number of possible ligand structures. With the binding site finalized, GNINA docking suite was then used to computationally model UR778Br-–receptor interactions. MD equilibration was done prior to the MD simulation. Both of these were simulated at a temperature of 298K and 1 bar of pressure. The equilibration process was conducted and the simulation was conducted for 3 nanoseconds. Images were generated by the libraries within the notebook.


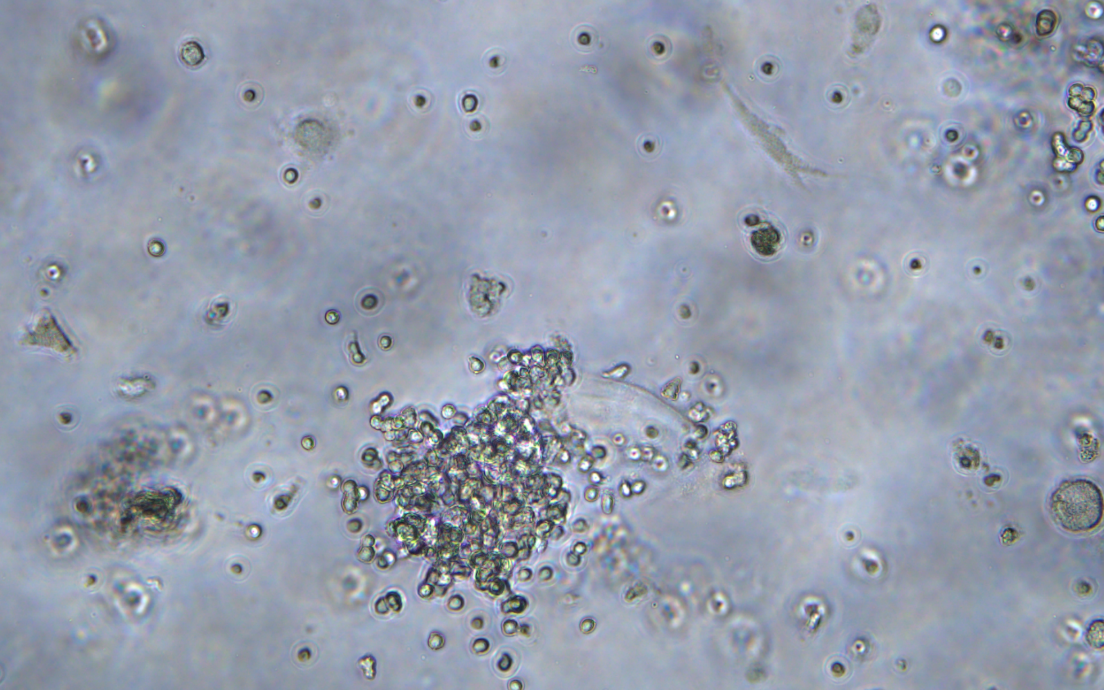


**
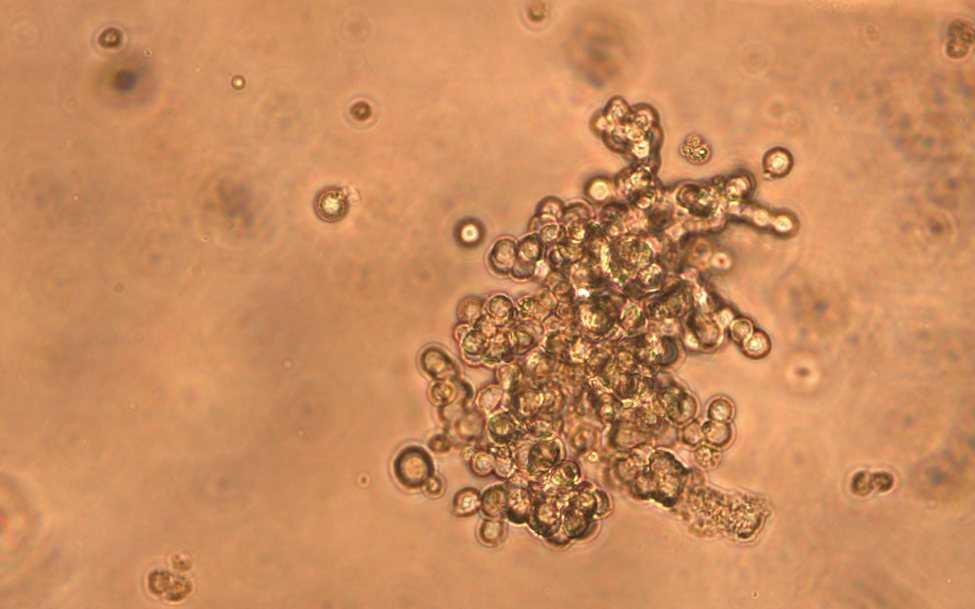
**

**Supplementary Figure-5:** Two representative CFU at day 15 primary AML3 exposed to DMSO.

**Supplementary Figure 6:**

Effect of UR778Br on MOLM13 and U937 (left), of the four analogs on MOLM13 (middle) and U937 (right). The four analogs had no effect of viability of MOLM13 and U937.

**A**

| **Sl No.** | **Ligands** | **Potential Energy-OPLS_2005 (kJ/mol)** | **Electrostatic Energy–OPLS_2005 (kJ/mol)** | **Van der Waal Energy-OPLS_2005 (kJ/mol)** |
| --- | --- | --- | --- | --- |
| **1.** | *(S)-*AK778 | -83643.852 | -64764.297 | -5163.775 |
| **2.** | *(S)-*UR778Br | -83707.055 | -65215.629 | -5142.294 |
| **3.** | *(R)-*AK778 | -83689.711 | -64956.965 | -5198.425 |
| **4.** | *(R)-*UR778Br | -83694.070 | -64989.297 | -5207.301 |

**B**

| **Sl No.** | **Ligands** | **Interacting residues** | **Type of Residues** | **Glide gscore**  **(docking score) kcal/mol** | **Number of**  **hydrophobic contacts** |
| --- | --- | --- | --- | --- | --- |
| 1. | *(S)-*AK778 | Thr 1008 | Hydrophilic | -4.797 | 12 |
|  |  | Ser 1298 | Polar |  |  |
|  |  | Tyr 1012 | Hydrophobic |  |  |
| 2. | *(S)-*UR778Br | Tyr1296,Tyr 1012 | Hydrophobic | -5.271 | 22 |
|  |  | Glu 1301 | Hydrophilic |  |  |
| 3. | *(R)-AK778* | Thr 10008 | Hydrophilic | -4.241 | 17 |
|  |  | Asn 1011 | Polar |  |  |
|  |  | Tyr 1296,Tyr 1012 | Hydrophobic |  |  |
| 4. | *(R)-*UR778Br | Tyr 1296 | Hydrophobic | -4.846 | 20 |
|  |  | Thr 10008 | Hydrophilic |  |  |

**C**

| **Sl No.** | **Ligands** | **Potential Energy-OPLS_2005 (kJ/mol)** | **Electrostatic Energy–OPLS_2005 (kJ/mol)** | **Van der Waal Energy-OPLS_2005 (kJ/mol)** |
| --- | --- | --- | --- | --- |
| **1.** | *(S)-*AK778 | -83609.797 | -65058.496 | -5170.973 |
| **2.** | *(S)-*UR778Br | -83699.898 | -65618.094 | -5160.767 |
| **3.** | *(R)-*AK778 | -83629.414 | -65337.309 | -5175.186 |
| **4.** | *(R)-*UR778Br | -83645.258 | -65312.621 | -5179.796 |

**D**

| **Sl No.** | **Ligands** | **Interacting residues** | **Type of Residues** | **Glide gscore**  **(docking score) kcal/mol** | **Number of**  **hydrophobic contacts** |
| --- | --- | --- | --- | --- | --- |
| 1. | *(S)-*AK778 | Glu 1078, Lys 1144 | Hydrophilic | -4.712 | 28 |
|  |  | Asn 1137 | Polar |  |  |
|  |  | Ala 1140, Leu1085,  Val 1141, Val 1075, Phe1145. | Hydrophobic |  |  |
| 2. | *(S)-*UR778Br | Lys 1144, Glu 1078 | Hydrophilic | -6.079 | 40 |
|  |  | Met 1138,Phe1145,  Val 1076, Leu 1085,  Val 1075, Ala 1140 | Hydrophobic |  |  |
| 3. | *(R)-AK778* | Glu 1078 | Hydrophilic | -4.174 | 23 |
|  |  | Leu 1085, Ile 1079,  Val 1075, Ala 1140,  Phe 1145, Leu 1085,  Val 1141 | Hydrophobic |  |  |
|  |  | Ser 1084, Asn 1137, Asn 1086 | Polar |  |  |
| 4. | *(R)-*UR778Br | Asn 1137, Ser 1134,Ser 1084,Asn 1086 | Polar | -4.723 | 23 |
|  |  | Asp 1082 | Hydrophilic |  |  |

**Supplementary Table-1:** (A-B): Energy calculations and docking results of the receptor-ligand complexes at the binding site around TRS. (C-D): Energy calculations and docking results of the receptor-ligand complexes at the top ranked site.

| **Sl No.** | **Ligands** | **Molecular Weight**  **(MW, Da)** | **Polar Surface Area(Å^2^)** | **logP_o/w_** | **logS** | **CNS** | **logBB** |
| --- | --- | --- | --- | --- | --- | --- | --- |
| 1. | *(S)*-AK778 | 339.39 | 85.643 | 3.793 | -4.117 | -2 | -1.012 |
| 2. | *(S)-*UR778Br | 418.30 | 87.678 | 4.425 | -5.377 | -1 | -0.982 |
| 3. | *(R)*-AK778 | 339.39 | 89.960 | 3.792 | -4.218 | -2 | -1.083 |
| 4. | *(R)-*UR778Br | 418.30 | 89.958 | 4.384 | -5.078 | -1 | -0.909 |

**Supplementary Table-2:** Physicochemical characteristics of (R and S- AK778) and (R and S-UR778Br. Among UR778Br analogs, R-isomer predicts superior LogP, PSA and LogS values.

FC-HS5-UR778Br

| 24h | Trypan Blue stain: manual cell count | | | 48h | Trypan Blue stain: manual cell count | | |
| --- | --- | --- | --- | --- | --- | --- | --- |
| Trypsin | µM UR778Br | Total cells | % Viability | Trypsin | µM UR778Br | Total cells | % Viability |
|  | 25 | 7.4x10^5^ | 99 |  | 25 | 8x10^5^ | 91 |
|  | 12.5 | 7.3x10^5^ | 99 |  | 12.5 | 7.9x10^5^ | 96 |
|  | 6.25 | 7.0x10^5^ | 99 |  | 6.25 | 7.9x10^5^ | 96 |
|  | 3.12 | 8.6x10^5^ | 99 |  | 3.12 | 8.6x10^5^ | 98 |
|  | DMSO | 9.0x10^5^ | 99 |  | DMSO | 8.6x10^5^ | 99 |
| Supernatant | µM UR778Br | Total cells | % Viability | Supernatant | µM UR778Br | Total cells | % Viability |
|  | 25 | 1.3x10^5^ | 50 |  | 25 | 0.5x10^5^ | 19 |
|  | 12.5 | 1.2x10^5^ | 75 |  | 12.5 | 0.6x10^5^ | 33 |
|  | 6.25 | 0.6x10^5^ | 75 |  | 6.25 | 0.7x10^5^ | 30 |
|  | 3.12 | 0.6x10^5^ | 75 |  | 3.12 | 1.3x10^5^ | 60 |
|  | DMSO | 0.6x10^5^ | 73 |  | DMSO | 1.4x10^5^ | 58 |

**Supplementary Table-3:** Primary AML cells co-cultured with unirradiated HS-5 cells were exposed to a range of concentrations of UR778Br and DMSO for 48 hours before flow- cytometric analysis with Annexin V and propidium iodide to quantify apoptosis and necrosis. In order to rule out the possibility of the observed effect on primary AML cell being indirect due to the effect of UR778Br on HS-5, we quantified viability of the adherent HS-5 cells at 24- and 48 hours by Trypan Blue dye exclusion of the adherent HS-5 cells. As shown in Table 3 UR778Br had no noticeable effects on the viability of HS-5 cells.

**References:**

Sela M, Mozes E, Shearer GM, Karniely Y. Cellular aspects of the inverse relationship between the net charge of immunogens and of antibodies elicited. Proceedings of the National Academy of Sciences. 1970 Nov;67(3):1288-93)

Ambrose MA. The surface properties of cancer cells: a review. Cancer res. 1962 Jun; 22:525-48.

Mehrishi JN. Positively charged amino groups on the surface of normal and cancer cells. European Journal of Cancer (1965). 1970 Apr 1;6(2):127-37.

HN Eisen, S Belman, and ME Carsten. The reaction of 2,4-dinitrofluorobenzenesulfonic acid with free amino groups of proteins. J Amer chem Soc 75,4583-4585.

Prema S, Madyastha KR, Sahasrabudhe MB. Electrophoretic mobility of (a) normal human leukocytes, (b) fluorodinitrobenzene tagged normal human leukocytes and (c) human leukaemic cells. Indian J Cancer. 1977 Sep; 14(3): 200-5).

Sahasrabudhe MB, Prema S, Madyastha KR, Gollerkeri MP. Studies on the use of chemically tagged normal white blood cells as antigens for producing specific immune response against human leukemic cells. Indian Journal of Cancer. 1972 Jun 1;9(2):101-11 and SS Joshi PhD thesis University of Bombay 1978.

Sahasrabudhe MB, Prema S, Madyastha KR, Gollerkeri MP, Rao SS. Development of a specific anti-leukaemic serum for the treatment of leukaemia in clinics. Nature. 1971 Jul 16;232(5307):197-8.

Karande AA, Sahasrabudhe MB. Isolation of leukaemia specific antigenic macromolecular moiety from dinitrophenylated normal human leukocytes. (Proceedings of the Indian Academy of Sciences-Section B 1978 Jan (Vol. 87, pp. 1-8). Springer India.
